# Evaluation of Enamel Topography after Debonding Orthodontic Ceramic Brackets by Different Er,Cr:YSGG and Er:YAG Lasers Settings

**DOI:** 10.3390/dj8010006

**Published:** 2020-01-09

**Authors:** Marwan Hoteit, Samir Nammour, Toni Zeinoun

**Affiliations:** 1 Department of Orthodontics, Faculty of Dental Medicine, Lebanese University, Beirut 27798, Lebanon; 2 Department of Dental Science, Faculty of Medicine, University of Liege, Liege 4020, Belgium; S.Namour@ulg.ac.be; 3 Department of Oral and Maxillofacial Surgery, Faculty of Dental Medicine, Lebanese University, Beirut 27798, Lebanon; zeinountoni@gmail.com

**Keywords:** erbium laser, adhesion, debonding, shear bond strength, microcracks, enamel loss topography

## Abstract

In the last decade, the success of lasers in simplifying many dental procedures has heightened the need for research in the orthodontic field, in order to evaluate the benefits of laser-assisted ceramic brackets debonding. Conventional ceramic brackets removal delivers a high shear bond strength (SBS), which might lead to enamel damage. Nowadays, debonding ceramic brackets by Er:YAG laser seems a viable alternative technique; however, there is no data on the use of Er,Cr:YSGG in the literature. We aimed to evaluate the difference in enamel topography derived from different erbium laser settings used during debonding. One hundred and eighty bovine incisors teeth were randomly divided into fifteen experimental groups, according to different erbium laser settings using scanning methods. SBS testing was performed after debonding; stereomicroscopic and SEM analyses were done after cleaning the remaining adhesive so as to assess the incidence of enamel microcracks formation and enamel loss. There were no statistically significant differences between the proportions of teeth with normal enamel topography within the control group when compared with any of the Er:YAG groups. However, the proportion of teeth with a normal enamel topography in Er,Cr:YSGG was 4 W/20 Hz (83.3%) and in Er:YAG was 5 W/20 Hz (91.7%), which was statistically significantly higher than the control group (41.7%). The selection of erbium lasers’ optimal parameters during debonding influences the enamel topography. When considering the evaluation of both microscopic and statistical analyses, irradiation by Er:YAG (120 mJ/40 Hz) displayed a significant reduction in microcracks compared with conventional debonding, even though some microstructural changes in the enamel could be noted. Er,Cr:YSGG (4 W/20 Hz) respected the enamel topography the most out of the studied groups.

## 1. Introduction

The use of lasers has increased tremendously in all fields of dentistry. Different types of lasers currently serve as a supplement to or as a replacement for some conventional procedures, such as caries removal [1], gingivectomy, frenectomy, and surgical exposure of teeth. Research in the contemporary orthodontic field opened new horizons in the acceleration of tooth movement, pain control, and bone regeneration, as well as the adhesion of orthodontic brackets and debonding procedures [2].

With the increased interest of patients in aesthetics, the use of ceramic brackets is becoming more popular. The main risk associated with the use of these brackets is that they deliver a high shear bond strength (SBS) at the time of debonding, which can cause a permanent cracking and tearing of the enamel [3,4,5]. This SBS depends mainly on the type of etching, the resin adhesive strength, and the nature of the enamel surface and the debonding techniques [3]. 

Erbium-family lasers offer several advantages in debonding orthodontic brackets because of their versatility, where changing the settings (water/air concentration, power, energy, frequency, time, and the irradiation method) would help to protect the enamel surface and prevent increasing the intrapulpal chamber temperature [6]. Zach and Cohen (1965) observed an absence of adverse effects on pulp tissue when the intrapulpal temperature increased by up to 1.8 °C [7]. Naltbangil et al. (2018) found that when debonding ceramic brackets by Er:YAG 2 W and 4 W for 6 s, the pulp chamber temperature did not increase by more than 0.67 ± 0.12 °C and 1.25 ± 0.16 °C 4 W, respectively, [8]. The ceramic bracket laser irradiation methods also influence the pulp temperature variation. The following three different techniques have been described: high energy single pulse [9], circular scanning methods [10], and S-shaped scanning methods [8,10,11]. According to Oztopark et al., scanning the laser beam for 6 s through the orthodontic bracket surface prevents a single point temperature rise, and consequently decreases heat dissipation to the pulp [11]. A water cooling system helps the erbium lasers to temper the pulpal temperature’s increase [12].

Enamel topography remains a major concern after brackets debonding. The application of lasers seems to be effective in the debonding orthodontic ceramic brackets for several reasons, as follows: it helps to reduce enamel microcracks (EMC) formation compared with conventional manual debonding [6,13], and decreases the shear bond strength by disrupting the bond between the ceramic bracket and enamel mainly at the bracket/cement interface, leaving the majority of the adhesive on tooth surface. However, the assessment of enamel topography after resin clean-up is not elucidated in the literature.

Few studies have been performed using Er:YAG focused on SBS [9,14,15], pulp temperature [8,10,16], and adhesive remnants index (ARI) [8,17], while none that we could find were done on Er,Cr:YSGG. It would be of interest to evaluate the outcome of different settings for the two erbium lasers on enamel topography, as these two lasers may perform differently.

The main objective of this research is to find debonding settings for both Er,Cr:YSGG and Er:YAG lasers that allow for the removal of the polycrystalline orthodontic ceramic brackets without changing the enamel topography.

The null hypothesis is that the use of erbium lasers in debonding orthodontic ceramic brackets does not help to protect the enamel topography after conventional adhesive removal.

## 2. Materials and Methods

This study was approved by the Lebanese University Ethics Committee of Beirut–Lebanon (no. CUMEB/D130/01072018). A total of one hundred and eighty Bovine teeth were selected using a x10 magnification stereomicroscope (Motic SMZ 140-N2 LED) to ensure that the enamel surface was intact with no caries or fractures. The teeth were stored and decontaminated according to International Organization for Standardization, Testing of Adhesion to Tooth Structure (ISO/TS 11405:2015 Dentistry) [16]. The sample was randomly divided into fifteen experimental groups of twelve teeth each, according to different laser debonding settings.

The buccal surface of the teeth was etched with a 37% phosphoric acid gel for 30 s (s), rinsed abundantly with water for 20 s, and subsequently dried with an oil-free air spray until the enamel surface showed a dull and frosty appearance. A fine layer of Transbond XT bonding (3M Unitek, Monrovia, CA, USA) was then applied to the etched surface, and the adhesive pre-coated bracket (APC) Flash-free, 3M clarity advance ceramic brackets were bonded on the buccal surface. The adhesive was cured for 20 s (by 3M ESPE, Elipar^TM^ S10 LED curing light) with an output power of 1200 mW/cm^2^. The teeth were fixed in a cylindrical resin mold that fits inside the clamp of a universal testing machine (Ultradent Products, Inc., South Jordan, UT, USA).

Six groups were debonded using Er,Cr:YSGG of a 2780 nm wavelength (Waterlase MD, Biolase technology, Inc., Irvine, CA, USA) with a MX7 sapphire tip, and with a 0.7 mm beam diameter at the impact point. A non-contact type handpiece (Turbo) in H mode (60 microseconds pulse duration) was used under a 70% air and 30% water scanning method, perpendicular to the bracket surface at the focal point for 6 s. The groups were classified according to Er,Cr:YSGG laser settings as follows: Er,Cr:YSGG 3 W/20 Hz (38.96 J/cm^2^), Er,Cr:YSGG 3 W/40 Hz (19.48 J/cm^2^), Er,Cr:YSGG 4 W/20 Hz (51.95 J/cm^2^), Er,Cr:YSGG 4 W/40 Hz (25.97 J/cm^2^), Er,Cr:YSGG 5 W/20 Hz (64.93 J/cm^2^), and Er,Cr:YSGG 5 W/40 Hz (32.47 J/cm^2^).

Eight groups were debonded with a Er:YAG laser wavelength of 2940 nm (Fidelis; Fotona, Medical laser, Ljubljana, Slovenia) using 0.9 mm as a beam diameter at the impact point. A non-contact type handpiece (HO2) with super short pulse mode (SSP mode: 50 microseconds pulse duration) and under air/water spray (air: 6 mL/min, water: 32 mL/min) scanning method, perpendicular to the bracket surface at focal point for 6 s. The groups were classified according to the Er:YAG energy level, as follows: Er:YAG 80 mJ/20 Hz (12.58 J/cm^2^), Er:YAG 80 mJ/40 Hz (12.58 J/cm^2^), Er:YAG 100 mJ/20 Hz (15.72 J/cm^2^), Er:YAG 100 mJ/40 Hz (15.72 J/cm^2^), Er:YAG 120 mJ/20 Hz (18.83 J/cm^2^), Er:YAG 120 mJ/40 Hz (18.83 J/cm^2^), Er:YAG 140 mJ/20 Hz (22.01 J/cm^2^), Er:YAG 140 mJ/40 Hz (22.01 J/cm^2^). The group control was conventionally debonded.

A universal testing machine (Ultradent Products, Inc., South Jordan, UT, USA) was used to measure the shear bond strength for all of the samples after laser debonding. A stereomicroscope analysis was done to assess the occurrence of EMC and enamel loss after the conventional cleaning of the remaining adhesive with a twelve-blade carbide bur. Three teeth from each group were analyzed by scanning electron microscopy (SEM; Seron technologies, Inc. Korea), at 100 to 1000× magnification.

After irradiation with the laser, the mechanical shear test was done with a chisel edge oriented vertically in an occluso-gingival direction and perpendicular to the bracket area base. The crosshead speed of the debonding machine was fixed at 1.0 mm/min. The SBS results were scored in Pound for each sample, then switched to Newton. The SBS values were converted to MPa using shear forces in Newton, and an upper incisor bracket area base (13.69 mm^2^).

### Statistical Analysis

Descriptive statistics were generated for the continuous variable of shear bond strength (SBS) and the following categorical variables: (1) incidence of enamel microcracks (EMC) (yes/no), (2) incidence of enamel loss, and (3) incidence of normal enamel topography (yes/no) where no EMC and enamel loss were detected. The statistics were generated across the three main test interventions ((1) Er,Cr:YSGG, (2) Er:YAG, and (3) control) and across the 15 test groups ((1) groups 1–6 (Er,Cr: YSGG), (2) groups 7–14 (Er:YAG), and (3) group 15 (control)).

To assess the associations between the incidence of normal enamel topography and the 15 test groups, Fisher’s test was used and was followed with post-hoc comparisons of the proportions. The tests were adjusted for all pairwise comparisons using the Bonferroni correction.

The mean SBS values were compared between the 15 test groups using one-way analysis of variance (ANOVA) tests followed by post-hoc comparisons. Welch’s ANOVA and Games–Howell post-hoc *p*-values were analyzed, as the assumption of homogeneity of variances was violated (Levene’s test *p*-value of <0.05). The IBM^®^ SPSS^®^ statistics version 20.0 statistical package was used to carry out all of the statistical analyses. Statistical significance was set at 0.05.

## 3. Results

### 3.1. Microscopic Analysis

#### 3.1.1. Stereomicroscopic Analysis

**Figure 1:**
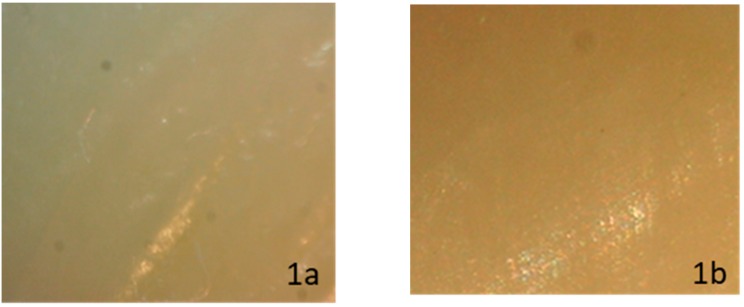
Stereomicroscopic evaluation showing no visible enamel topography changes before ceramic bracket bonding (**1a**) and after laser debonding (**1b**). The enamel conserved its normal glazed appearance.

**Figure 2:**
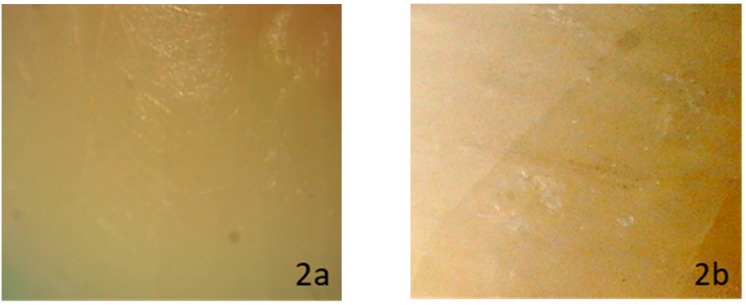
Stereomicroscopic evaluation showing formation of enamel microcracks after laser debonding (**2b**) from a previously sound enamel surface (**2a**).

**Figure 3:**
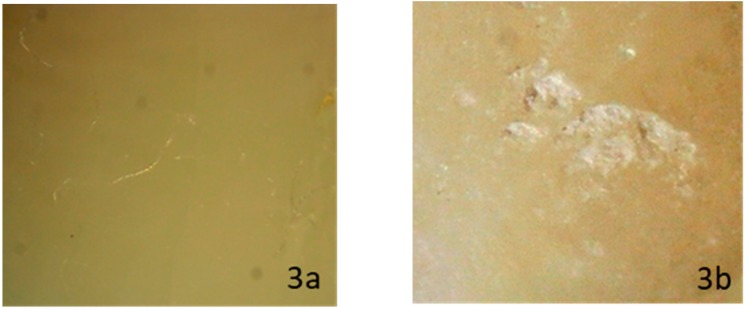
Stereomicroscopic evaluation shows sound enamel surface before ceramic bracket bonding (**3a**), while it reveals visible area of localized enamel loss after laser debonding (**3b**).

**Figure 4:**
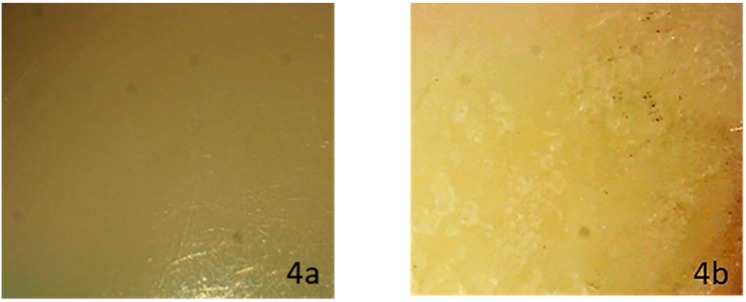
Stereomicroscopic evaluation shows sound enamel surface before ceramic bracket bonding (**4a**), while it shows scattered enamel loss after laser debonding (**4b**).

When evaluating enamel topography after ceramic bracket debonding and conventional resin removal with twelve blade carbide bur, observed under a ×10 magnification stereomicroscope, none of the subgroups showed a specific appearance. The enamel surface varied between normal glazed enamel (Figure 1b), or damaged enamel topography, which could appear as the presence of additional EMC (Figure 2b), localized enamel loss (Figure 3b), and scattered enamel loss (Figure 4b) underneath the debonded bracket area (Supplementary Material S1).

Within the Er,Cr:YSGG group, only the group debonded with 3 W/20 Hz had teeth that developed EMC post debonding (41.7%), whereas more than half of the control group (58.3%) developed microcracks (Table 1). On the other hand, all Er:YAG groups had teeth that developed microcracks, except for the group treated with 120 mJ/40 Hz. The percentage of teeth with microcracks among the Er:YAG groups ranged from 8.3% for the group of 100 mJ/40 Hz to 50% for the group of 120 mJ/20 Hz (Table 1).

There were incidents of enamel loss in all laser-treated groups. For the Er,Cr:YSGG groups, the incidence ranged between 8.3% and 83.3% whereas for Er:YAG groups the incidence ranged between 41.7% and 100% (Table 1). None of the control teeth experience enamel loss of any severity on the stereomicroscopic study at 10× magnification.

**Figure 5:**
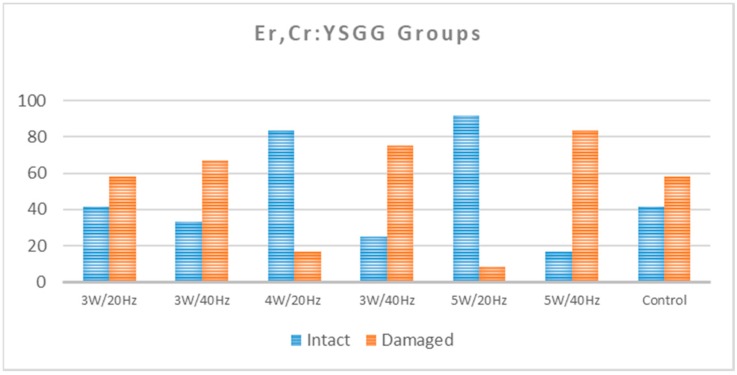
Shows the percentage of intact and damaged enamel in each of the Er,Cr:YSGG groups compared to the control group.

**Figure 6:**
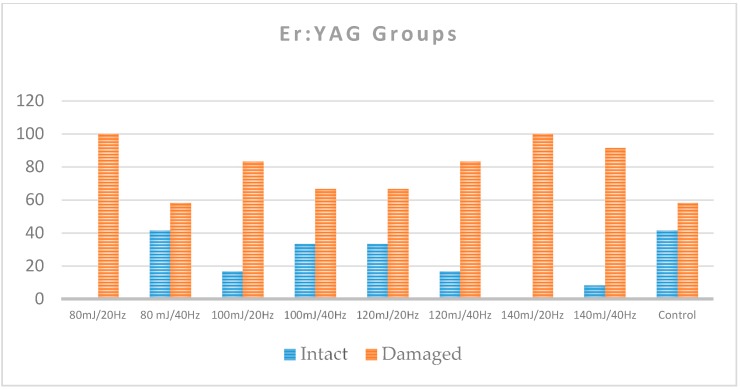
Shows the percentage of intact and damaged enamel in each of the Er:YAG groups compared to the control group.

As assessed by the occurrence of either EMC or enamel loss after the debonding of ceramic brackets, 41.7% of the control teeth remained with a normal enamel topography. Among the Er,Cr:YSGG groups, the percentage of intact teeth ranged from 16.7% to 91.7%, with the lowest percentages for 5 W/40 Hz and the highest percentages for 4 W/20 Hz and 5 W/20 Hz (Table 1 and Figure 5). Among the Er:YAG groups, the lowest percentages of intact teeth were for groups 80 mJ/20 Hz, 140 mJ/20 Hz, and 140 mJ/40 Hz (0%, 0%, and 8.3%, respectively), and the highest group was 80 mJ/40 Hz (41.7%; Table 1 and Figure 6).

There was a statistically significant association between the prevalence of intact enamel and the 15 study groups (*p* < 0.001; Table 2).

In the Er,Cr:YSGG groups, the proportion of teeth with a normal enamel topography was statistically significantly smaller in the control group (41.7%) when compared to the groups of 4 W/20 Hz (83.3%) and 5 W/20 Hz (91.7%; *p* < 0.05; Table 2 and Table 3). In the comparison between the control group and the groups of 3 W/20 Hz, 4 W/40 Hz, 5 W/40 Hz, and 3 W/40 Hz, there were no statistically significant differences (*p* > 0.05). However, there were no statistically significant differences between the proportions of teeth with intact enamel within the control group when compared with any of the Er:YAG groups (groups 7–14; *p* > 0.05; Table 3).

#### 3.1.2. Scanning Electron Microscope Analysis after Debonding

**Figure 7:**
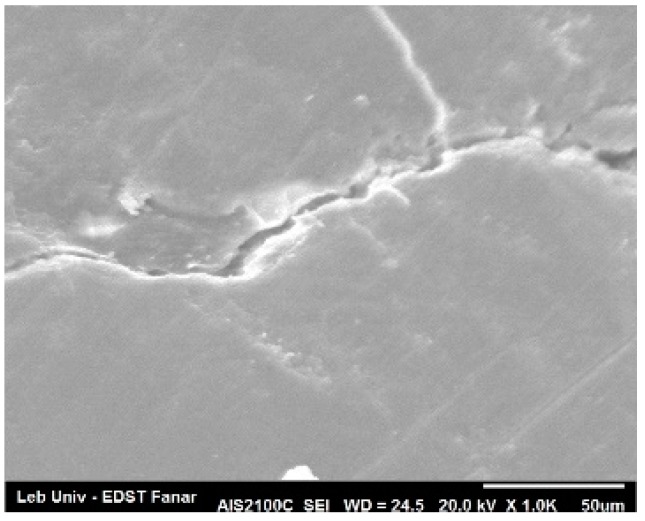
Control group.

In the control group, the enamel surface showed extended microcracks formation, enamel tearing, and localized small areas of enamel loss (Figure 7).

**Figure 8:**
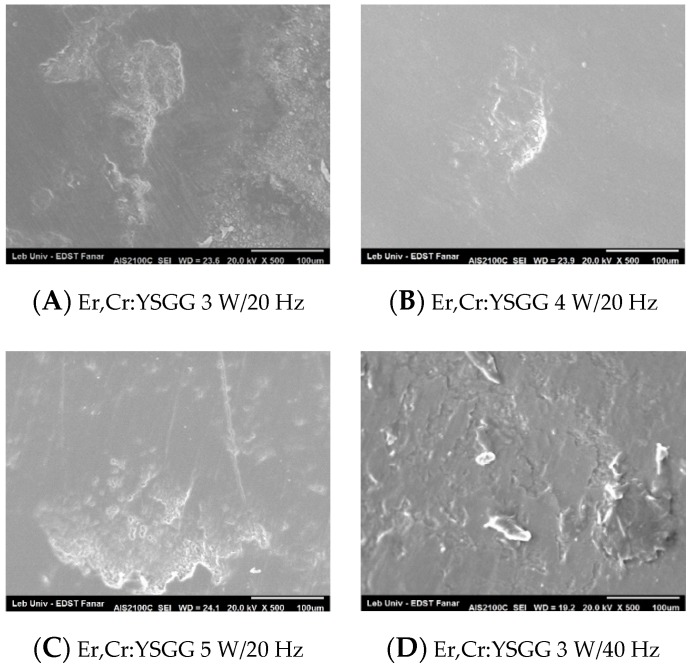

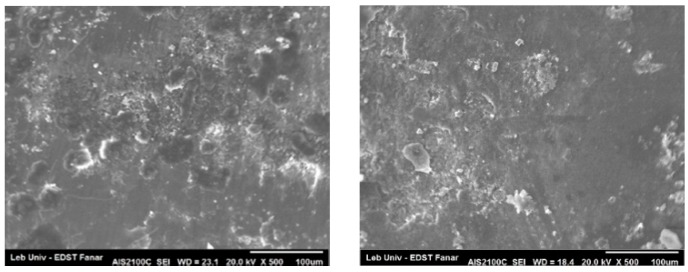
SEM figures of enamel topography debonded by Er,Cr:YSGG subgroups.

**Figure 9:**
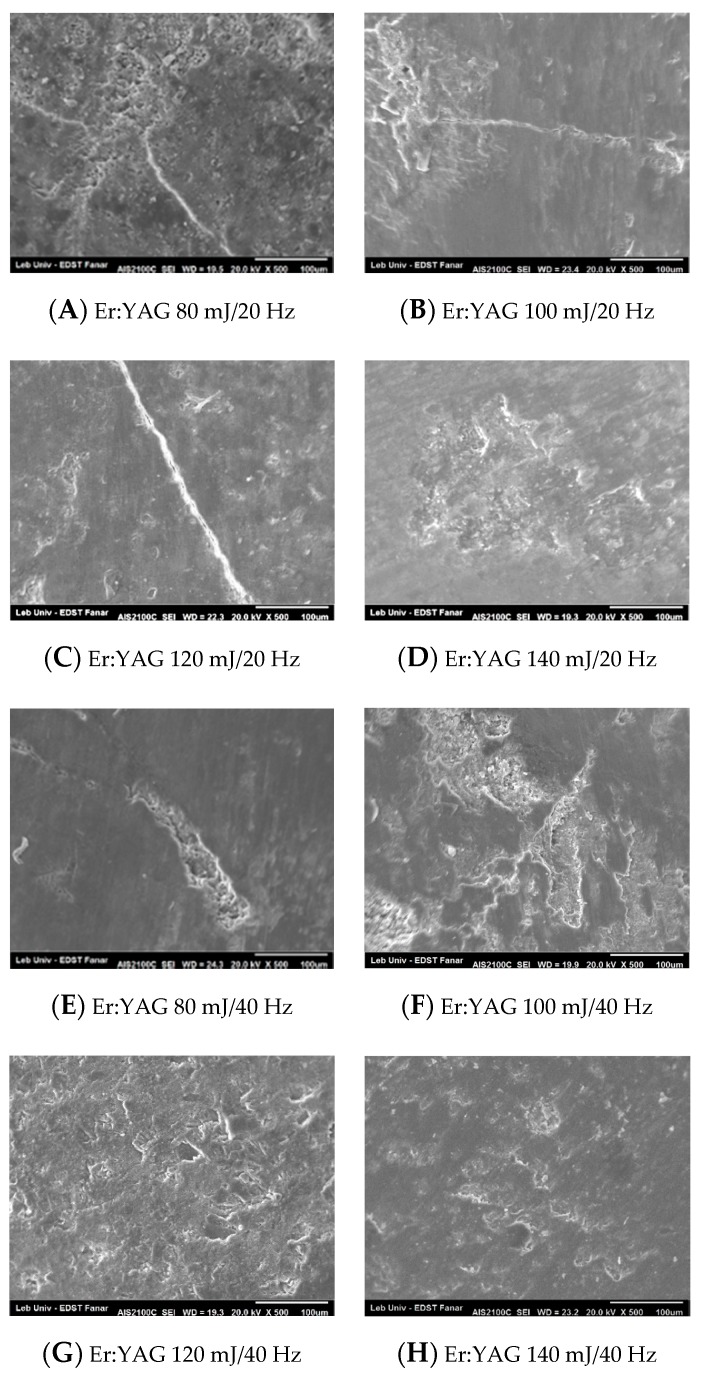
SEM figures of enamel topography debonded by Er:YAG subgroups.

The enamel topography evaluated by SEM after debonding ceramic brackets with Er,Cr:YSGG laser confirm the stereomicroscopic study. The groups of 4 W/20 Hz and 5 W/20 Hz showed a predominant homogeneous enamel surface (Figure 8B,C). On the other hand, the groups of 3 W/20 Hz, 3 W/40 Hz, 4 W/40 Hz, and 5 W/40 Hz revealed different degrees of scattered enamel loss (Figure 8A,D–F). When debonding was done with Er:YAG settings of 80 mJ/20 Hz, the enamel displayed an irregular porous appearance with EMC formation, and a localized area of enamel loss could also be observed (Figure 9A). Group Er:YAG 80 mJ/40 Hz showed a localized detached enamel area and scattered enamel loss (Figure 9E). Groups Er:YAG 100 mJ/20 Hz and Er:YAG 120 mJ/20 Hz presented a heteregeneous enamel surface with enamel loss and EMC formation (Figure 9B,C). The SEM figures of the group debonded with Er:YAG 100 mJ/40 Hz settings presented structural change with areas of extensive enamel loss, surrounded by an irregularly damaged enamel surface (Figure 9F), while the groups debonded with Er:YAG 120 mJ/40 Hz, Er:YAG 140 mJ/20 Hz, and 140 mJ/40 Hz exhibited an amorphous stripped enamel surface appearance with flattened shiny changes (Figure 9G,D,H; Supplementary Material S2).

### 3.2. Analytical Results

#### Shear Bond Strength

The mean shear bond strength (SBS) levels ranged between 5.30 ± 5.26 MPa (Er,Cr:YSGG 5 W/20 Hz), and 21.07 ± 1.80 MPa (control group; Table 4). The minimum recorded SBS level was 0.00 (groups Er,Cr:YSGG 3 W/20 Hz and 5 W/20 Hz, and Er:YAG 100 mJ/40 Hz, 120 mJ/40 Hz, and 140 mJ/40 Hz) and the maximum was 29.72 (Er:YAG 80 mJ/20 Hz). All of the control group teeth exhibited SBS values larger than 13 MPa (Table 4). For groups Er,Cr:YSGG 4 W/20 Hz and 5 W/20 Hz only, 50% or more of the teeth recorded SBS values of <8 MPa. For groups Er,Cr:YSGG 3 W/40 Hz, 4 W/40 Hz, 5 W/40 Hz, and Er:YAG 120 mJ/20 Hz and 140 mJ/20 Hz, none of the teeth had SBS values of <8 MPA.

In the comparison of mean SBS values between the 15 individual test groups (*p* < 0.001; Table 5), there were statistically significant differences in the mean SBS values between the group control and all other groups, except Er:YAG groups 80 mJ/20 Hz, 100 mJ/20 Hz, 120 mJ/20 Hz, and 80 mJ/40 Hz (*p* ≥ 0.106; Table 5). All of the Er,Cr:YSGG groups showed statistically significant smaller mean SBS values than the control group (*p* ≤ 0.037). Similarly, mean SBS values for the Er:YAG groups of 100 mJ/40 Hz, 120 mJ/40 Hz, 140 mJ/40 Hz, and 140 mJ/20 Hz were also statistically significantly smaller than for the control group (*p* ≤ 0.006).

## 4. Discussion

The main purpose of debonding orthodontic ceramic brackets by lasers is to reduce the adhesion of the resin to the bracket base. In fact, after laser irradiation, bond failure will occur mostly at the bracket–resin interface or within the resin, in contrary to conventional debonding where the failure is more likely at the resin–enamel interface [8]. Bond failure, after irradiating the bracket surface with lasers, could be explained by the thermomechanical ablation that occurs in the superficial part of the adhesive layer [6]. In this study, the selection of Er:YAG and Er,Cr:YSGG lasers was because erbium family lasers are highly absorbed by the remnant water present in the adhesive resin layer. However, Er:YAG and Er,Cr:YSGG lasers are both absorbed by enamel hydroxyapatite, which has a greater affinity to Er:YAG [15]. Therefore, the risk of enamel changes after irradiation should be considered. The various laser parameters used for the debonding procedure, such as laser wavelength, emission mode, power density, time of exposure, water/air irrigation, and the use of a scanning method, can help to protect the enamel topography and to reduce the enamel damage compared to conventional manual debonding [8]. It is estimated that in the case of Er:YAG, polycrystalline ceramic brackets transmit 85% of the energy applied to the surface that reaches the resin adhesive [9]. However, no studies have been done on an Er,Cr:YSGG laser for debonding orthodontic ceramic brackets from the enamel surface.

### 4.1. Enamel Topography

To study the enamel topography after debonding the ceramic bracket, EMC and enamel loss were analyzed under stereomicroscope and SEM after adhesive clean up.

Group control showed a high percentage (58.3%) of EMC, some of which were ramified and extended apical and incisal to the bracket area, as described also by Dumbryte [18,19]. This group displayed a minor area of enamel loss that appeared only on the SEM observations. Sorel noted also a small area, with less than 10% of damaged enamel after manual debonding [20].

The Er,Cr:YSGG subgroups did not show any microcracks formation after ceramic bracket debonding, except in the 3 W/20 Hz group. When analyzing the enamel topography, the two groups that showed statistically significant intact enamel were Er,Cr:YSGG 4 W/20 Hz (83.3%) and 5 W/20 Hz (91.7%). Enamel loss, when present on the stereomicroscope, was minor and localized only under the bracket. This was confirmed on the SEM observation, as these two groups showed the most homogenous enamel topography between all of the tested groups, even though the enamel surface for the group of 4 W/20 Hz was more uniform than for the group of 5 W/20 Hz. We should note that no studies have been performed on Er,Cr:YSGG for the debonding of the orthodontic ceramic bracket from enamel that permits comparison with the literature (Supplementary Material S1 and S2).

For the groups debonded with Er:YAG, all of the subgroups presented changes in enamel topography. The subgroups irradiated with Er:YAG 100 mJ/40 Hz and 120 mJ/40 Hz displayed a reduced EMC incidence when compared to the control (8.3%, 0%, and 58.3%, respectively). However, when evaluating the number of teeth with normal enamel topography, these two subgroups failed to exhibit statistically significant differences with the control group (33.3%, 16.7%, and 41.7%, respectively). The reason was that under stereomicroscopic study, the enamel surface looked irregular, and on SEM, the enamel topography looked rough and amorphous in the group of 100 mJ/40 Hz, while the group of 120 mJ/40 Hz revealed stripped and shiny changes. Our results concerning the incidence of EMC in groups Er:YAG 100 mJ/40 Hz and 120 mJ/40 Hz are in agreement with the published literature, as the EMC was statistically significantly reduced [6,8,9,10]. When scanning polycrystalline ceramic brackets with 170 mJ/20 Hz, Grzech-Leśniak et al. described images of a typical enamel surface treated with Er:YAG laser with minor thermal damage in a prism structure [10]. Nalbantgil et al., when irradiating with 140 mJ/20 Hz, found no volcano-like hollows on the enamel surface [17]. Dostalova et al., using Er:YAG 280 mJ/6 Hz, observed no damage to the enamel [6]. Additionally, Mundethu et al., when operating with a single pulse of 600 mJ/2 Hz, for a 800 μs pulse duration, confirmed the same results on the cross-sectional SEM images [9]. Our results agreed with those of Grzech-Leśniak et al., as the presence of minor damages under the bracket area was confirmed. The diversity in enamel topographies appearance could be due to the different protocols used during the debonding polycrystalline ceramic brackets. We should note that, even in our study, various laser settings yielded to different enamel topography after debonding. To our knowledge, most studies assessed the quality of the enamel after debonding ceramic brackets with a Er:YAG laser using the adhesive remnant index (ARI), where when the debonding site gets closer to the enamel–adhesive interface, the risk of damage enamel surface increases. The hallmark of our study was assessing the enamel topography by the direct observation of the enamel surface, which could give a clinically significant assessment by the direct evaluation of the post-laser debonding enamel topography.

### 4.2. Shear Bond Strength

**Figure 10:**
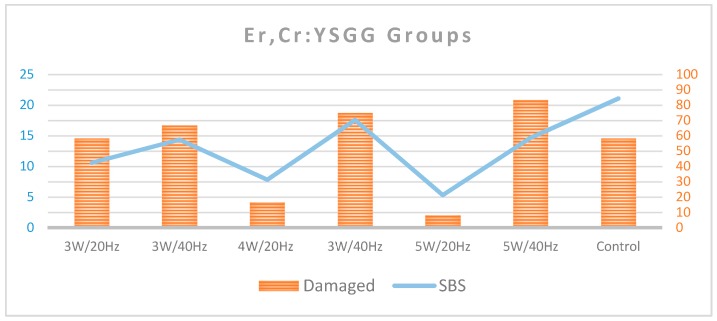
Summarize the mean SBS value percentages and the percentage of damaged enamel in each of the Er,Cr:YSSG groups.

The control group presented the highest SBS mean values of 21.07 ± 1.80 MPa, and were all larger than 13 MPa. The mean SBS levels for the groups of Er,Cr:YSGG 4 W/20 Hz and 5 W/20 Hz ranged between 7.8 ± 3.95 MPa and 5.30 ± 5.26 MPa, respectively, where 50% or more of the teeth recorded SBS values of <8 MPa and only 8.3% of group Er,Cr:YSGG 5 W/20 Hz exceeded 13 MPa. The percentage of samples showing a damaged enamel surface in groups Er,Cr:YSGG 4 W/20 Hz and 5 W/20 Hz are 16.7% and 8.3%, respectively (Figure 10).

**Figure 11:**
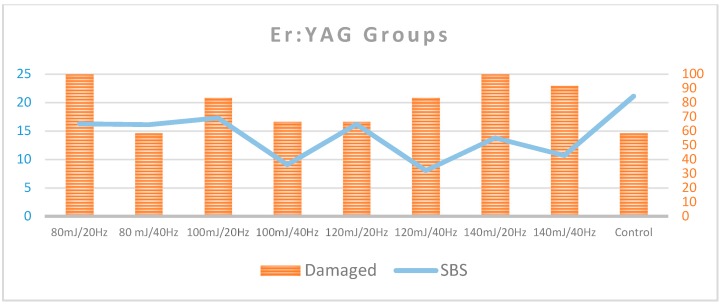
Summarize the mean SBS value percentages and the percentage of damaged enamel in each of the Er:YAG groups.

In the Er:YAG groups, even though the group of 120 mJ/40 Hz showed a SBS mean value around 8 MPa and all its samples remained below 13 MPa, the percentage of enamel damage reached 83.3% (Figure 11).

In the literature, there is no agreement on the clinically accepted SBS values that prevent enamel damage while debonding orthodontic brackets from the enamel surface. When evaluating conventional orthodontic bracket debonding, Reynolds (1975) considered that 6 to 8 MPa could be the minimum clinically acceptable SBS while debonding orthodontic brackets [21]. Endo et al. (2009) found that values of SBS between 2.80 and 10.00 MPa did not cause damage or enamel loss [22]. Rodriguez-Chavez et al. (2017) found that even with SBS values as low as 6.8 MPa, it is possible to observe damage and enamel loss [23]. Enamel tearing could be observed when SBS values were higher than 13.8 MPa [24].

Recently, Mirhashemi et al. did not show a decrease in the SBS values of ceramic brackets bonded on composite blocks after Er,Cr:YSGG laser irradiation with a power of 3 W and an energy density of 22–28 J/cm^2^, and concluded that this may be due to the interaction of the laser beam with two similar substrates, the resin and the composite [25]. With regard to SBS values, the Er:YAG 120 mJ/40 Hz subgroup presented similar results as described in the literature [8,14,17]. However, Nalbantgil et al., using a higher energy of 200 mJ/20 Hz, succeeded to reduce the mean SBS value to 3.28 MPa ± 0.73 [5], which was below those found in our study.

When trying to explain the previous observations in the different groups, it should be considered that a change in enamel topography after debonding could be multifactorial and not only related to debonding forces [26]. During laser debonding, the target substrate should be the water present in the resin. After being scattered inside the polycrystalline ceramic bracket, the laser beam would be first partially absorbed by the remnant water in the resin monomer, and then by both the water and hydroxyapatite present in the enamel, which might consequently produce undesirable enamel damage.

Ideally, the setting of the laser applied should fulfill the following two conditions: (1) at the resin level, the laser beam should be totally absorbed by the water remnants of the adhesive resin, resulting in the degradation of its matrix. This would reduce the ceramic bracket SBS, facilitating debonding without causing additional stress on the enamel surface. (2) At the enamel level, the remaining beam reaching the enamel should neither cause ablation of the surface nor disturb the mechanical properties of the enamel.

It seems when using lasers to debond ceramic brackets, the remaining beam reaching enamel might change the enamel microstructure according to the energy level that could be ablative [10], or even subablative, in both erbium family lasers. The risk of enamel irradiation under the bracket while debonding with Er:YAG has been previously described [10], and it could increase at the thinnest bracket area, especially around its borders and the slot. Consequently, this would cause the permanent alteration of enamel topography under the bracket area that remained rough even after conventionally cleaning the adhesive resin.

Different enamel topographies were detected on the SEM in the bracket debonded area. It ranged from normal enamel topography, surfaces etched enamel, enamel crystallization, enamel loss, or even zones of enamel ablation. The final enamel topography was the outcome of the amount of energy absorbed by the enamel, the resulting change of its mechanical properties, and the applied shear forces. It should be noted that when Er,Cr:YSGG [27] or Er:YAG [28] increases enamel microhardness, fracture toughness will decrease, as these two measures are inversely proportional [29]. This may lead to a more brittle enamel, where the risk of enamel microcracks formation and enamel loss is increased when debonding ceramic brackets.

## 5. Conclusions

Within the limitations of our study, it can be concluded that the improper adjustment of laser parameters may alter the enamel surface during ceramic bracket debonding.

As the texture of enamel topography was altered, further studies should assess the enamel microstructural changes, as well as the mechanical properties of the enamel, like microhardness and fracture toughness.

The null hypothesis was partially rejected, as, compared to conventional manual debonding, the use of Er,Cr:YSGG (4 W/20 Hz) in debonding orthodontic ceramic brackets helped to protect the enamel topography after conventional adhesive removal. The improper adjustment and manipulation of erbium lasers’ debonding settings could be related to the structural alteration of the enamel beneath the bracket and its surroundings area.

## Figures and Tables

**Table 1:** Distribution sample by enamel status post debonding (n = 180).

Group			Modified Enamel Topography		Normal Enamel Topography
			EMC	Enamel Loss	
			n (%)	n (%)	n (%)
**Er,Cr:YSGG**	1	3 W/20 Hz	5 (41.7)	4 (33.3)	5 (41.7)
	2	3 W/40 Hz	0 (0.0)	8 (66.6)	4 (33.3)
	3	4 W/20 Hz	0 (0.0)	2 (16.7)	10 (83.3)
	4	4 W/40 Hz	0 (0.0)	9 (75.0)	3 (25.0)
	5	5 W/20 Hz	0 (0.0)	1 (8.3)	11 (91.7)
	6	5 W/40 Hz	0 (0.0)	10 (83.3)	2 (16.7)
**Er:YAG**	7	80 mJ/20 Hz	4 (33.3)	10 (83.3)	0 (0.0)
	8	80 mJ/40 Hz	5 (41.7)	5 (41.7)	5 (41.7)
	9	100 mJ/20 Hz	2 (16.7)	10 (83.3)	2 (16.7)
	10	100 mJ/40 Hz	1 (8.3)	7 (58.3)	4 (33.3)
	11	120 mJ/20 Hz	6 (50.0)	6 (50.0)	4 (33.3)
	12	120 mJ/40 Hz	0 (0.0)	10 (83.3)	2 (16.7)
	13	140 mJ/20 Hz	3 (25.0)	12 (100)	0 (0.0)
	14	140 mJ/40 Hz	2 (16.7)	11 (91.7)	1 (8.3)
**Control**	15	Conventional	7 (58.3)	0 (0.0)	5 (41.7)

Notes. SBS—shear bond strength; groups 1–6: Er,Cr:YSGG; groups 7–14: Er:YAG; group 15: control group (conventional debonding). EMC—enamel microcracks.

**Table 2:** Association between the test group and the presence of intact enamel after debonding (n = 180).

Group			Presence of Intact Enamel		Fisher’s Exact Test	
			Damaged n (%)	Intact n (%)	Test Statistic	*p* Value
**Er,Cr:YSGG**	1	3 W/20 Hz	7 (58.3)	5 (41.7)	52.730	<0.001 *
	2	3 W/40 Hz	8 (66.7)	4 (33.3)		
	3	4 W/20 Hz	2 (16.7)	10 (83.3)		
	4	4 W/40 Hz	9 (75)	3 (25.0)		
	5	5 W/20 Hz	1 (8.3)	11 (91.7)		
	6	5 W/40 Hz	10 (83.3)	2 (16.7)		
**Er:YAG**	7	80 mJ/20 Hz	12 (100.0) ^a^	0 (0.0) ^b^		
	8	80 mJ/40 Hz	7 (58.3)	5 (41.7)		
	9	100 mJ/20 Hz	10 (83.3)	2 (16.7)		
	10	100 mJ/40 Hz	8 (66.7)	4 (33.3)		
	11	120 mJ/20 Hz	8 (66.7)	4 (33.3)		
	12	120 mJ/40 Hz	10 (83.3)	2 (16.7)		
	13	140 mJ/20 Hz	12 (100.0) ^a^	0 (0.0) ^b^		
	14	140 mJ/40 Hz	11 (91.7)	1 (8.3)		
**Control**	15	Conventional	7 (58.3)	5 (41.7)		

Notes. Groups 1–6: Er,Cr:YSGG; groups 7–14: Er:YAG; group 15: control group (conventional debonding). ^a^ Transformed to count = 11 for comparisons of column proportions; ^b^ Transformed to count = 1 for comparisons of the column proportions. * Statistically significant at *p* < 0.01.

**Table 3:** Statistically significant post-hoc pairwise comparisons for the association between the presence of normal enamel topography and laser (n = 180).

	Significant Post-Hoc Pairwise Comparisons ^a^		
	Laser Group		
	Groups 1, 2, 4, 6, 7, 8, 9, 10, 11, 12, 13, and 14	Groups 3,5	Group Control
Normal topography		1, 2, 4, 6, 7, 8, 9, 10, 11, 12, 13, 14, and 15	
Damaged topography	3 and 5		3 and 5

Notes. Groups 1–6: Er,Cr:YSGG; groups 7–14: Er:YAG; group 15: control group (conventional debonding). For each significant pair, the number of the category with the smaller column proportion appears under the category with the larger column proportion. ^a^ Tests are adjusted for all pairwise comparisons using the Bonferroni correction.

**Table 4:** Descriptive statistics for shear bond strength (SBS; n = 180). SD—standard deviation.

Group		SBS						
		Mean	SD	(Min.; Max.)	Coeff. Var. (%)	<8 n (%)	8–13 n (%)	>13 n (%)
**Er,Cr:YSGG**	3 W/20 Hz	10.57	5.18	(0.00; 19.11)	49.01	3 (25.0)	5 (41.7)	4 (33.3)
	3 W/40 Hz	14.35	2.17	(10.10; 17.3)	15.12	0 (0.0)	4 (33.3)	8 (66.7)
	4 W/20 Hz	7.80	3.95	(1.53; 12.80)	50.64	6 (50.0)	6 (50.0)	0 (0.0)
	4 W/40 Hz	17.56	2.47	(13.80; 20.4)	14.07	0 (0.0)	0 (0.0)	12 (100.0)
	5 W/20 Hz	5.30	5.26	(0.00; 16.60)	99.25	8 (66.7)	3 (25.0)	1 (8.3)
	5 W/40 Hz	14.65	3.96	(10.64; 21.0)	27.03	0 (0.0)	6 (50.0)	6 (50.0)
**Er:YAG**	80 mJ/20 Hz	16.24	9.14	(5.83; 29.72)	56.28	4 (33.3)	2 (16.7)	6 (50.0)
	80 mJ/40 Hz	16.09	5.34	(4.87; 22.03)	33.19	2 (16.7)	0 (0.0)	10 (83.3)
	100 mJ/20 Hz	17.27	9.35	(3.90; 29.17)	54.14	3 (25.0)	1 (8.3)	8 (66.7)
	100 mJ/40 Hz	9.06	5.21	(0.00; 18.60)	57.51	4 (33.3)	5 (41.7)	3 (25.0)
	120 mJ/20 Hz	16.14	4.44	(9.46; 21.11)	27.51	0 (0.0)	4 (33.3)	8 (66.7)
	120 mJ/40 Hz	8.02	4.36	(0.00; 12.89)	54.36	5 (41.7)	7 (58.3)	0 (0.0)
	140 mJ/20 Hz	13.77	3.57	(8.01; 18.60)	25.93	0 (0.0)	6 (50.0)	6 (50.0)
	140 mJ/40 Hz	10.68	6.36	(0.00; 18.11)	59.55	5 (41.7)	1 (8.3)	6 (50.0)
**Conventional debonding**		21.07	1.80	(17.69; 24.2)	8.54	0 (0.0)	0 (0.0)	12 (100.0)

**Table 5:** Distribution of SBS by various test groups (n = 180). ANOVA—analysis of variance.

Group				SBS					ANOVA Test					
				Mean		SD			Test Statistic ^a^			*p* Value		
**Er,Cr:YSGG**	3 W/20 Hz			10.57		5.18			18.395			<0.001 **		
	3 W/40 Hz			14.35		2.17								
	4 W/20 Hz			7.80		3.95								
	4 W/40 Hz			17.56		2.47								
	5 W/20 Hz			5.30		5.26								
	5 W/40 Hz			14.65		3.96								
Er:YAG	80 mJ/20 Hz			16.24		9.14								
	80 mJ/40 Hz			16.09		5.34								
	100 mJ/20 Hz			17.27		9.35								
	100 mJ/40 Hz			9.06		5.21								
	120 mJ/20 Hz			16.14		4.44								
	120 mJ/40 Hz			8.02		4.36								
	140 mJ/20 Hz			13.77		3.57								
	140 mJ/40 Hz			10.68		6.36								
Conventional debonding				21.07		1.80								
**Games–Howell Post-Hoc Comparisons (*p* Value)**														
**Control**	**1**	**2**	**3**	**4**	**5**	**6**	**7**	**8**	**9**	**10**	**11**	**12**	**13**	**14**
	0.001 *	0.037 *	<0.001 **	0.006 **	<0.001 **	<0.001 **	0.006 **	<0.001 **	**0.106**	**0.973**	<0.001 **	**0.242**	**0.862**	0.001 **

Notes. SBS: Shear bond strength; SD: standard deviation. ^a^ Welch’s robust ANOVA reported since Levene’s test of homogeneity of variances *p* value <0.001. * Statistically significant at *p* < 0.05; ** statistically significant at *p* < 0.01.

## Supplementary Materials

The following are available online at https://www.mdpi.com/2304-6767/8/1/6/s1.
