# Supplementary material for: Evaluation of Enamel Topography after Debonding Orthodontic Ceramic Brackets by Different Er,Cr:YSGG and Er:YAG Lasers Settings
Source: Dent J (Basel). 2020 Jan 9;8(1):6. doi: 10.3390/dj8010006 (PMC7175227; doi:10.3390/dj8010006)
Supplement: Supplementary file 1 [file dentistry-08-00006-s001.zip › Supplementary file/figures and schemes.docx]

File for figures and schemes

**Stereomicroscopic analysis**

| **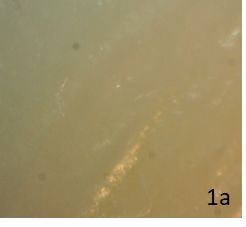** | **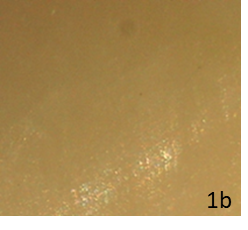** |
| --- | --- |
| **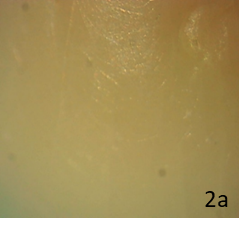** | **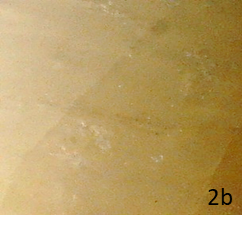** |
| **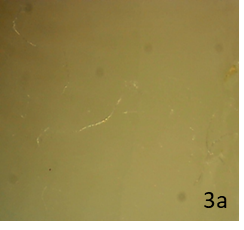** | **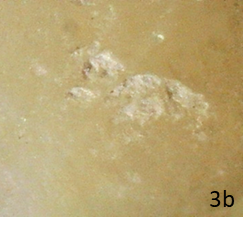** |
| **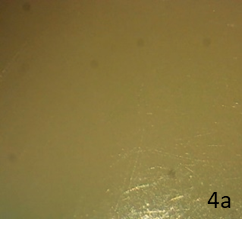** | **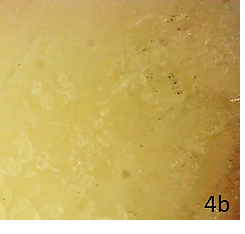** |

Figure (1-4): Stereomicroscopic evaluation— Enamel topography sample after laser debonding. Figures (1a-2a-3a-4a) show sound enamel surface before ceramic bracket bonding. Figure (1b) normal glazed enamel, figure (2b) enamel microcracks, figure (3b) localized enamel loss, figure (4b) scattered enamel loss.

Figure 5: shows the percentage of intact and damaged enamel in each of the Er,Cr:YSGG groups compared to control group

Figure 6: shows the percentage of intact and damaged enamel in each of the Er:YAG groups compared to control group

**Scanning electron microscope analysis after debonding**

| **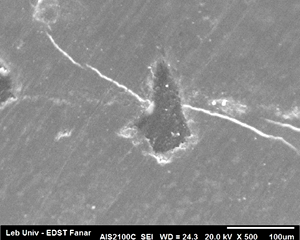** |
| --- |
| **Figure 7: Control group** |

| **Figure 6. SEM figures of enamel topography debonded by Er,Cr:YSGG subgroups** | | |
| --- | --- | --- |
| 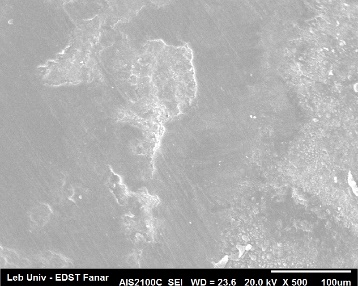 | 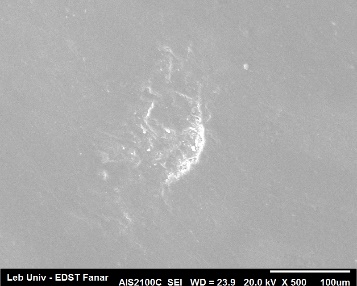 | 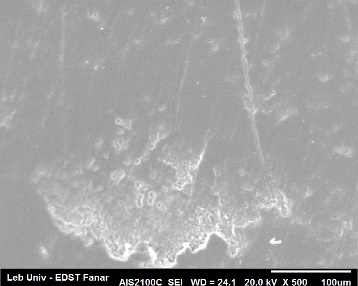 |
| Figure 8A) Er,Cr:YSGG 3W/20Hz | Figure 8B) Er,Cr:YSGG 4W/20Hz | Figure 8C) Er,Cr:YSGG 5W/20Hz |
| 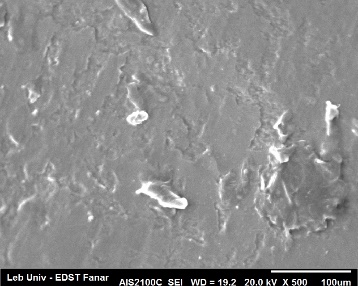 | 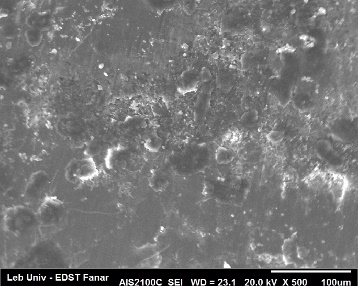 | **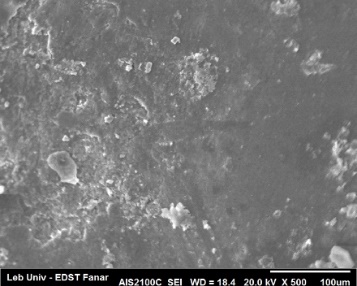** |
| Fiure 8D) Er,Cr:YSGG 3W/40Hz | Figure 8E) Er,Cr:YSGG 4W/40Hz | Figure 8F) Er,Cr:YSGG 5W/40Hz |

| **Figure 7. SEM figures of enamel topography debonded by Er:YAG subgroups** | | | |
| --- | --- | --- | --- |
| 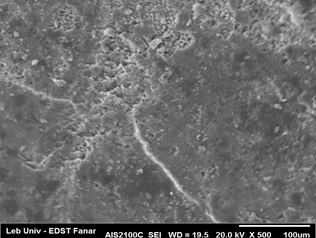 | 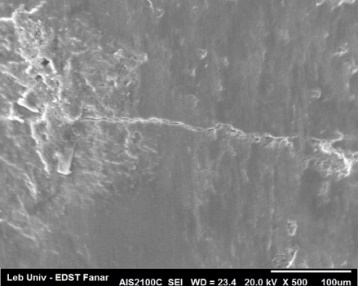 | **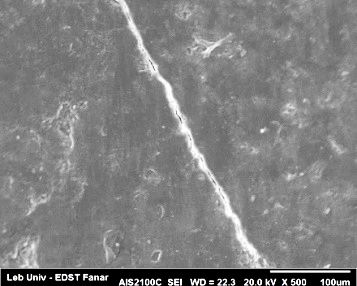** | **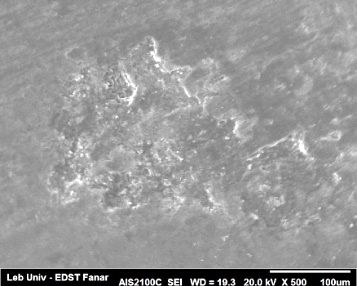** |
| Figure 9A) Er:YAG 80mJ/20Hz | Figure 9B) Er:YAG 100mJ/20Hz | Figure 9C) Er:YAG 120mJ/20Hz | Figure 9D) Er:YAG 140mJ/20Hz |
| 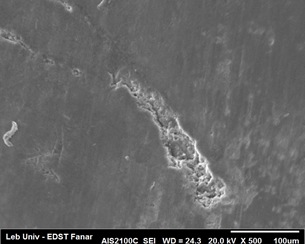 | 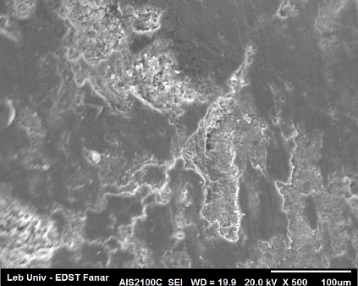 | 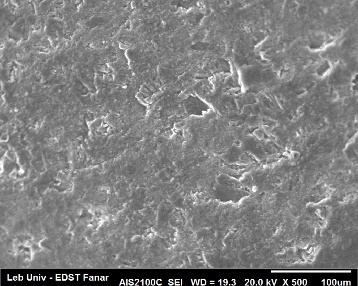 | 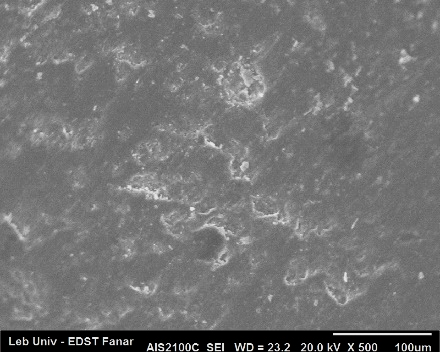 |
| Figure 9E) Er:YAG 80mJ/40Hz | Figure 9F) Er:YAG 100mJ/40Hz | Figure 9G) Er:YAG 120mJ/40Hz | Figure 9H) Er:YAG 140mJ/40Hz |

Figure 10: summarize the mean SBS values percentage and the percentage of damaged enamel in each of the Er,Cr:YSGG groups

Figure 11: summarize the mean SBS values percentage and the percentage of damaged enamel in each of the Er:YAG groups

**Table 1.** Distribution sample by enamel status post debonding (N=180)

| **Group** | | | **Modified enamel topography** | | **Normal enamel topography** |
| --- | --- | --- | --- | --- | --- |
|  |  |  | **EMC** | **Enamel loss** |  |
|  |  |  | **n (%)** | **n (%)** | **n (%)** |
| **Er,Cr:YSGG** | 1 | 3W/20Hz | 5 (41.7) | 4 (33.3) | 5 (41.7) |
|  | 2 | 3W/40Hz | 0 (0.0) | 8 (66.6) | 4 (33.3) |
|  | 3 | 4W/20Hz | 0 (0.0) | 2 (16.7) | 10 (83.3) |
|  | 4 | 4W/40Hz | 0 (0.0) | 9 (75.0) | 3 (25.0) |
|  | 5 | 5W/20Hz | 0 (0.0) | 1 (8.3) | 11 (91.7) |
|  | 6 | 5W/40Hz | 0 (0.0) | 10 (83.3) | 2 (16.7) |
| **Er:YAG** | 7 | 80mJ/20Hz | 4 (33.3) | 10 (83.3) | 0 (0.0) |
|  | 8 | 80mJ/40Hz | 5 (41.7) | 5 (41.7) | 5 (41.7) |
|  | 9 | 100mJ/20Hz | 2 (16.7) | 10 (83.3) | 2 (16.7) |
|  | 10 | 100mJ/40Hz | 1 (8.3) | 7 (58.3) | 4 (33.3) |
|  | 11 | 120mJ/20Hz | 6 (50.0) | 6 (50.0) | 4 (33.3) |
|  | 12 | 120mJ/40Hz | 0 (0.0) | 10 (83.3) | 2 (16.7) |
|  | 13 | 140mJ/20Hz | 3 (25.0) | 12 (100) | 0 (0.0) |
|  | 14 | 140mJ/40Hz | 2 (16.7) | 11 (91.7) | 1 (8.3) |
| **Control** | 15 | Conventional | 7 (58.3) | 0 (0.0) | 5 (41.7) |

Notes. SBS: Shear bond strength; Groups 1-6: Er,Cr:YSGG; Groups 7-14: Er:YAG; group 15: control group (conventional debonding).

**Table 2.** Association between test group and the presence of intact enamel after debonding (N=180)

| **Group** | | | **Presence of intact enamel** | | **Fisher’s Exact Test** | |
| --- | --- | --- | --- | --- | --- | --- |
|  |  |  | **Damaged**  **n (%)** | **Intact**  **n (%)** | **Test statistic** | **p value** |
| **Er,Cr:YSGG** | 1 | 3W/20Hz | 7 (58.3) | 5 (41.7) | 52.730 | <0.001^*^ |
|  | 2 | 3W/40Hz | 8 (66.7) | 4 (33.3) |  |  |
|  | 3 | 4W/20Hz | 2 (16.7) | 10 (83.3) |  |  |
|  | 4 | 4W/40Hz | 9 (75) | 3 (25.0) |  |  |
|  | 5 | 5W/20Hz | 1 (8.3) | 11 (91.7) |  |  |
|  | 6 | 5W/40Hz | 10 (83.3) | 2 (16.7) |  |  |
| **Er:YAG** | 7 | 80mJ/20Hz | 12 (100.0)^a^ | 0 (0.0)^b^ |  |  |
|  | 8 | 80 mJ/40Hz | 7 (58.3) | 5 (41.7) |  |  |
|  | 9 | 100mJ/20Hz | 10 (83.3) | 2 (16.7) |  |  |
|  | 10 | 100mJ/40Hz | 8 (66.7) | 4 (33.3) |  |  |
|  | 11 | 120mJ/20Hz | 8 (66.7) | 4 (33.3) |  |  |
|  | 12 | 120mJ/40Hz | 10 (83.3) | 2 (16.7) |  |  |
|  | 13 | 140mJ/20Hz | 12 (100.0)^a^ | 0 (0.0)^b^ |  |  |
|  | 14 | 140mJ/40Hz | 11 (91.7) | 1 (8.3) |  |  |
| **Control** | 15 | Conventional | 7 (58.3) | 5 (41.7) |  |  |

Notes. Groups 1-6: Er,Cr:YSGG; Groups 7-14: Er:YAG; group 15: control group (conventional debonding).

^a^Transformed to count = 11 for the purposes of comparisons of column proportions

^b^ Transformed to count = 1 for the purposes of comparisons of column proportions

^*^ Statistically significant at p < 0.01.

**Table 3.** Statistically significant post-hoc pairwise comparisons for the association between presence of normal enamel topography and laser (N=180)

|  | **Significant post-hoc pairwise comparisons^a^** | | |
| --- | --- | --- | --- |
|  | **Laser group** | | |
|  | **Groups 1, 2, 4, 6, 7, 8, 9, 10, 11, 12, 13, 14** | **Groups 3,5** | **Group control** |
| Normal topography |  | 1, 2, 4, 6, 7, 8, 9, 10, 11, 12, 13, 14, 15 |  |
| Damaged topography | 3,5 |  | 3,5 |

*Notes.* Groups 1-6: Er,Cr:YSGG; Groups 7-14: Er:YAG; group 15: control group (conventional debonding).

For each significant pair, the number of the category with the smaller column proportion appears under the category with the larger column proportion;

*^a^* tests are adjusted for all pairwise comparisons using the Bonferroni correction.

**Table 4.** Descriptive statistics for shear bond strength (SBS) (N=180)

| **Group** | | **SBS** | | | | | | |
| --- | --- | --- | --- | --- | --- | --- | --- | --- |
|  |  | **Mean** | **SD** | **(Min.; Max.)** | **Coeff. Var. (%)** | **< 8**  **n (%)** | **8-13**  **n (%)** | **>13**  **n (%)** |
| **Er,Cr:YSGG** | 3W/20Hz | 10.57 | 5.18 | (0.00; 19.11) | 49.01 | 3 (25.0) | 5 (41.7) | 4 (33.3) |
|  | 3W/40Hz | 14.35 | 2.17 | (10.10; 17.3) | 15.12 | 0 (0.0) | 4 (33.3) | 8 (66.7) |
|  | 4W/20Hz | 7.80 | 3.95 | (1.53; 12.80) | 50.64 | 6 (50.0) | 6 (50.0) | 0 (0.0) |
|  | 4W/40Hz | 17.56 | 2.47 | (13.80;20.4) | 14.07 | 0 (0.0) | 0 (0.0) | 12 (100.0) |
|  | 5W/20Hz | 5.30 | 5.26 | (0.00; 16.60) | 99.25 | 8 (66.7) | 3 (25.0) | 1 (8.3) |
|  | 5W/40Hz | 14.65 | 3.96 | (10.64;21.0) | 27.03 | 0 (0.0) | 6 (50.0) | 6 (50.0) |
| **Er:YAG** | 80mJ/20Hz | 16.24 | 9.14 | (5.83; 29.72) | 56.28 | 4 (33.3) | 2 (16.7) | 6 (50.0) |
|  | 80 mJ/40Hz | 16.09 | 5.34 | (4.87; 22.03) | 33.19 | 2 (16.7) | 0 (0.0) | 10 (83.3) |
|  | 100mJ/20Hz | 17.27 | 9.35 | (3.90; 29.17) | 54.14 | 3 (25.0) | 1 (8.3) | 8 (66.7) |
|  | 100mJ/40Hz | 9.06 | 5.21 | (0.00; 18.60) | 57.51 | 4 (33.3) | 5 (41.7) | 3 (25.0) |
|  | 120mJ/20Hz | 16.14 | 4.44 | (9.46; 21.11) | 27.51 | 0 (0.0) | 4 (33.3) | 8 (66.7) |
|  | 120mJ/40Hz | 8.02 | 4.36 | (0.00; 12.89) | 54.36 | 5 (41.7) | 7 (58.3) | 0 (0.0) |
|  | 140mJ/20Hz | 13.77 | 3.57 | (8.01; 18.60) | 25.93 | 0 (0.0) | 6 (50.0) | 6 (50.0) |
|  | 140mJ/40Hz | 10.68 | 6.36 | (0.00; 18.11) | 59.55 | 5 (41.7) | 1 (8.3) | 6 (50.0) |
| **Conventional debonding** | | 21.07 | 1.80 | (17.69;24.2) | 8.54 | 0 (0.0) | 0 (0.0) | 12 (100.0) |

| **Table 5:** Distribution of SBS by various test groups (N=180) | | | | | | | | | | | | | | | | | |
| --- | --- | --- | --- | --- | --- | --- | --- | --- | --- | --- | --- | --- | --- | --- | --- | --- | --- |
| **Group** | | | | **SBS** | | | | | **ANOVA Test** | | | | | | | | |
|  |  |  |  | **Mean** | | **SD** | | | **Test statistic^a^** | | | **p value** | | | | | |
| Er,Cr:YSGG | 3W/20Hz | | | 10.57 | | 5.18 | | | 18.395 | | | <0.001^**^ | | | | | |
|  | 3W/40Hz | | | 14.35 | | 2.17 | | |  |  |  |  |  |  |  |  |  |
|  | 4W/20Hz | | | 7.80 | | 3.95 | | |  |  |  |  |  |  |  |  |  |
|  | 4W/40Hz | | | 17.56 | | 2.47 | | |  |  |  |  |  |  |  |  |  |
|  | 5W/20Hz | | | 5.30 | | 5.26 | | |  |  |  |  |  |  |  |  |  |
|  | 5W/40Hz | | | 14.65 | | 3.96 | | |  |  |  |  |  |  |  |  |  |
| Er:YAG | 80mJ/20Hz | | | 16.24 | | 9.14 | | |  |  |  |  |  |  |  |  |  |
|  | 80mJ/40Hz | | | 16.09 | | 5.34 | | |  |  |  |  |  |  |  |  |  |
|  | 100mJ/20Hz | | | 17.27 | | 9.35 | | |  |  |  |  |  |  |  |  |  |
|  | 100mJ/40Hz | | | 9.06 | | 5.21 | | |  |  |  |  |  |  |  |  |  |
|  | 120mJ/20Hz | | | 16.14 | | 4.44 | | |  |  |  |  |  |  |  |  |  |
|  | 120mJ/40Hz | | | 8.02 | | 4.36 | | |  |  |  |  |  |  |  |  |  |
|  | 140mJ/20Hz | | | 13.77 | | 3.57 | | |  |  |  |  |  |  |  |  |  |
|  | 140mJ/40Hz | | | 10.68 | | 6.36 | | |  |  |  |  |  |  |  |  |  |
| Conventional debonding | | | | 21.07 | | 1.80 | | |  |  |  |  |  |  |  |  |  |
|  | | | **Games-Howell Post-Hoc comparisons (*p* Value)** | | | | | | | | | | | | | | |
| **Control** | **1** | **2** | **3** | | **4** | | **5** | **6** | **7** | **8** | **9** | **10** | **11** | **12** | **13** | **14** |  |
|  | **0.001^*^** | **0.037^*^** | **<0.001^**^** | | **0.006^**^** | | **<0.001^**^** | **<0.001^**^** | **0.006^**^** | **<0.001^**^** | 0.106 | 0.973 | **<0.001^**^** | 0.242 | 0.862 | **0.001^**^** |  |
