# Supplementary material for: Evaluation of Enamel Topography after Debonding Orthodontic Ceramic Brackets by Different Er,Cr:YSGG and Er:YAG Lasers Settings
Source: Dent J (Basel). 2020 Jan 9;8(1):6. doi: 10.3390/dj8010006 (PMC7175227; doi:10.3390/dj8010006)
Supplement: Supplementary file 1 [file dentistry-08-00006-s001.zip › Supplementary file/S 2 (SEM).docx]

Appendix of SEM

| **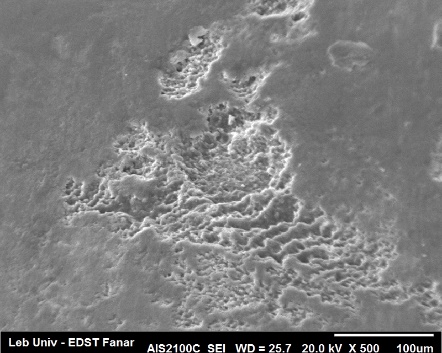** | 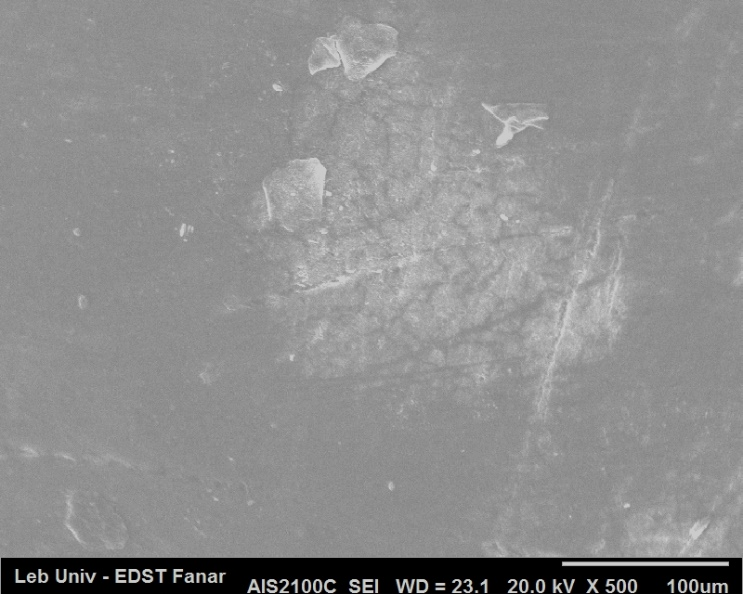 | 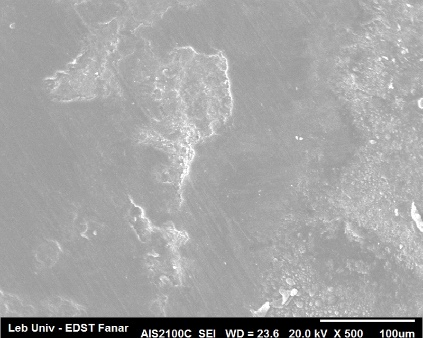 |
| --- | --- | --- |
| Fig. 6A) Er,Cr:YSGG 3W/20Hz | |  |
| 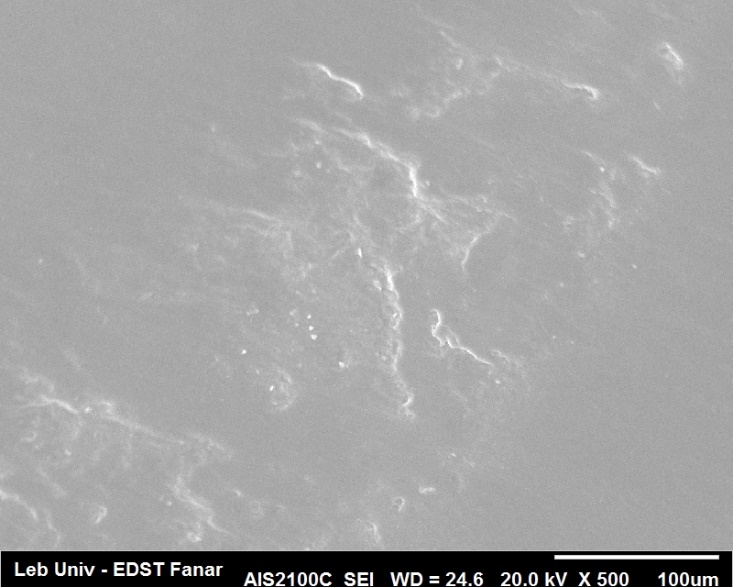 | 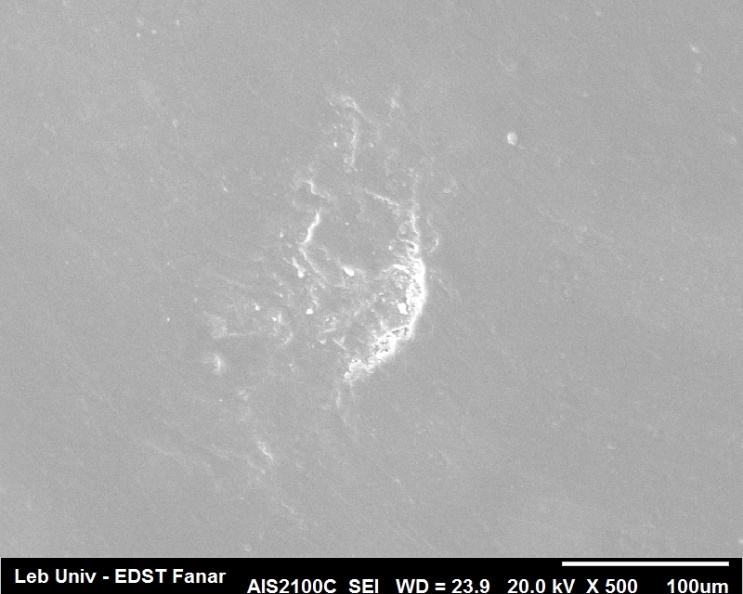 | 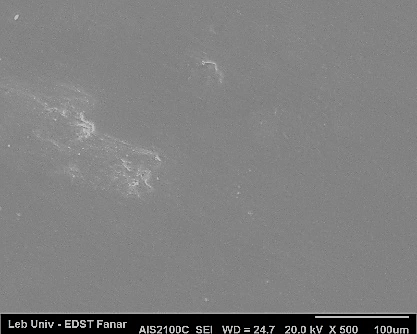 |
| **Fig. 6B) Er,Cr:YSGG 4W/20Hz** | |  |
| 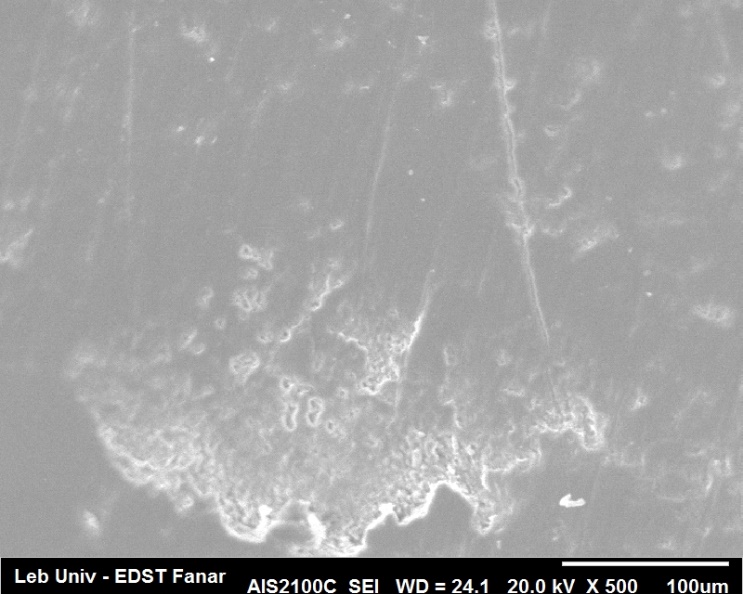 | 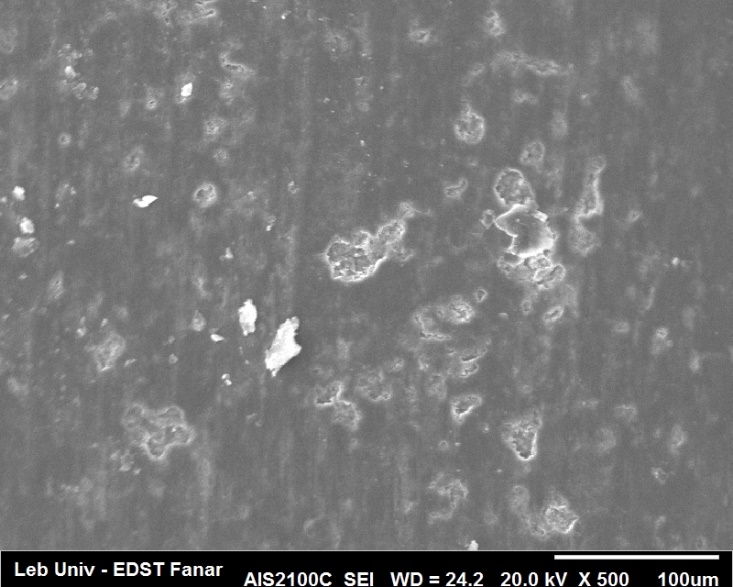 | **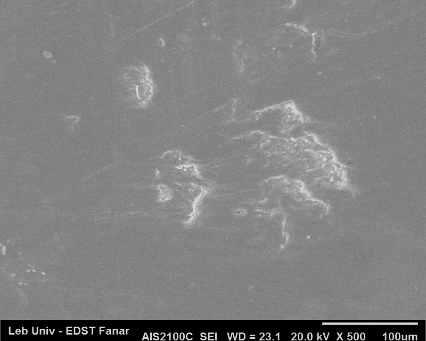** |
| Fig. 6C) Er,Cr:YSGG 5W/20Hz | |  |
| 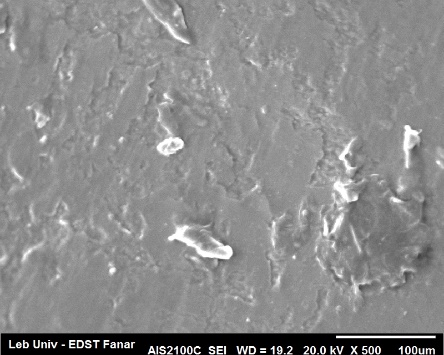 | 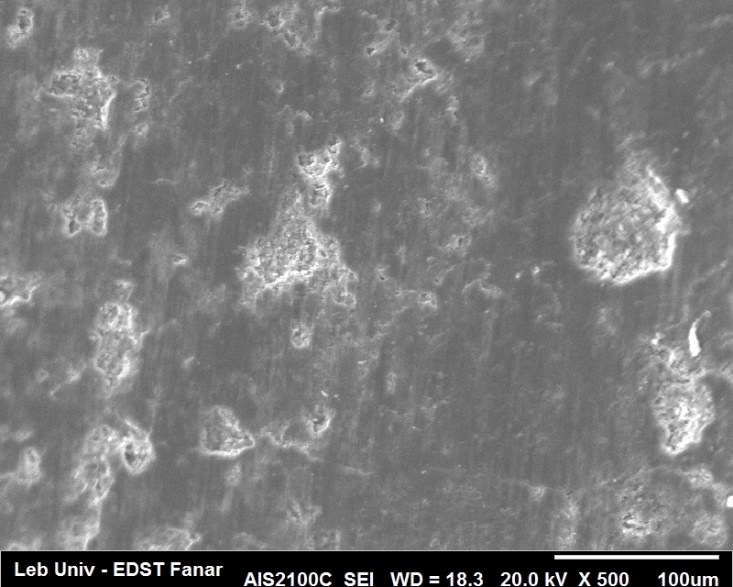 | 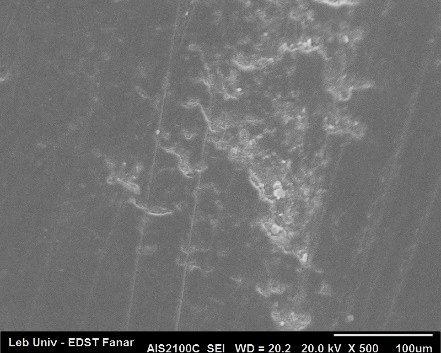 |
| **Fig. 6D) Er,Cr:YSGG 3W/40Hz** | |  |
| 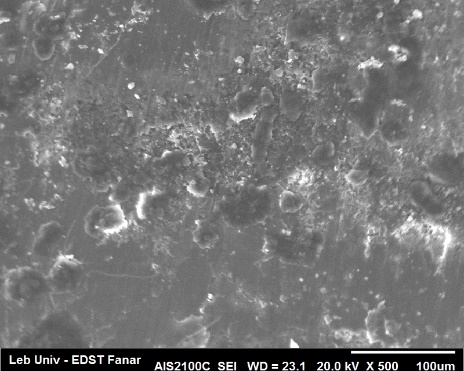 | 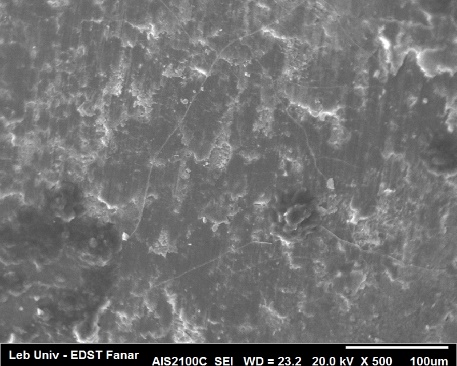 | 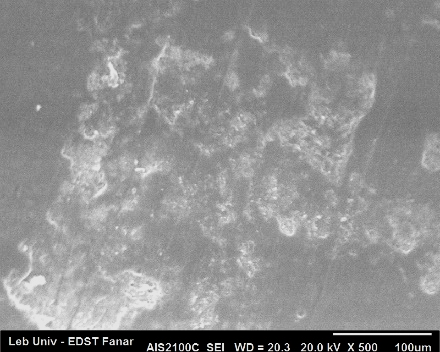 |
| **Fig. 6E) Er,Cr:YSGG 4W/40Hz** | |  |
| 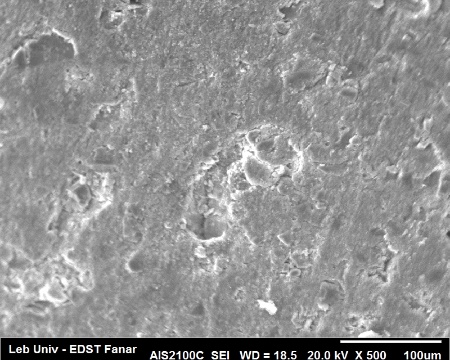 | 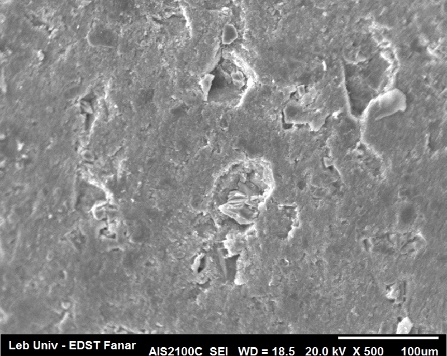 | 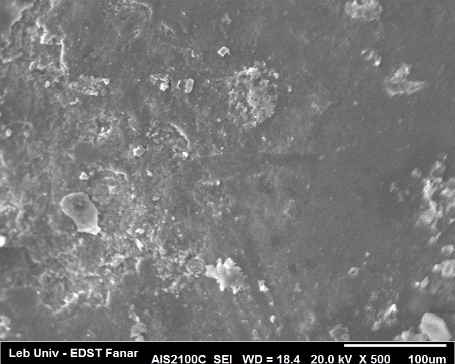 |
| **Fig. 6F) Er,Cr:YSGG 5W/40Hz** | |  |

| 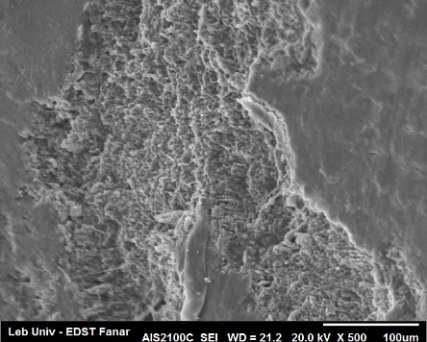 | 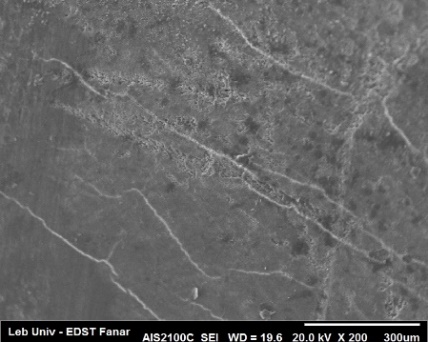 | 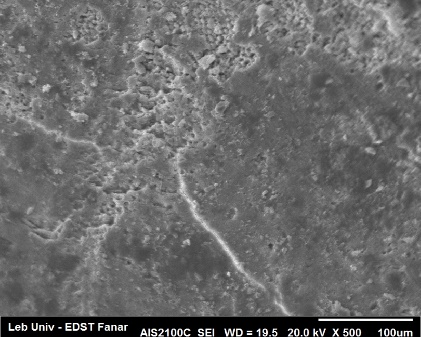 |
| --- | --- | --- |
| Fig. 7A) Er:YAG 80mJ/20Hz | |  |
| 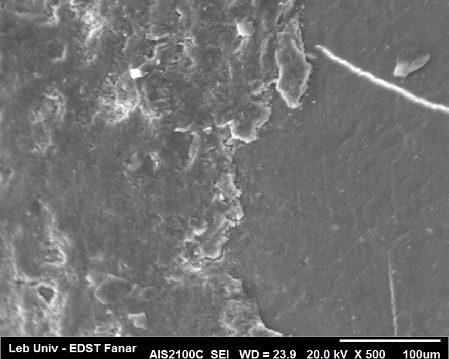 | 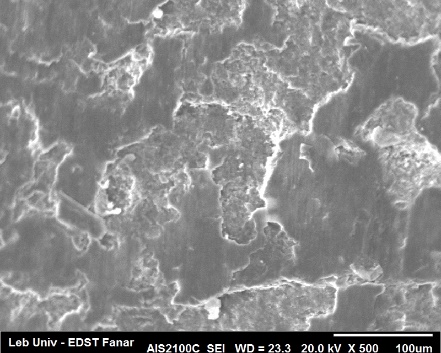 | 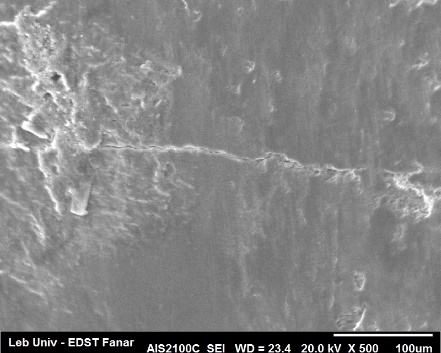 |
| Fig. 7B) Er:YAG 100mJ/20Hz | | |
| 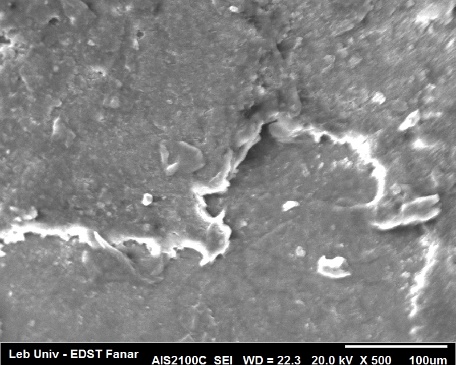 | 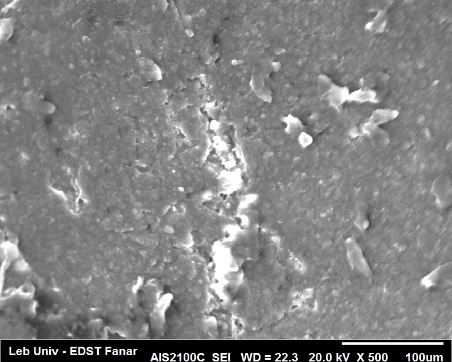 | 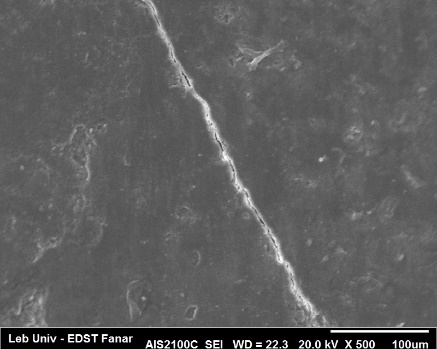 |
| 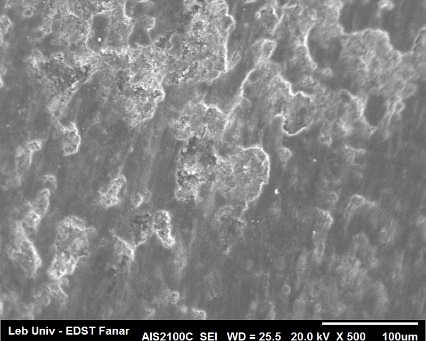Fig. 7C) Er:YAG 120mJ/20Hz | | 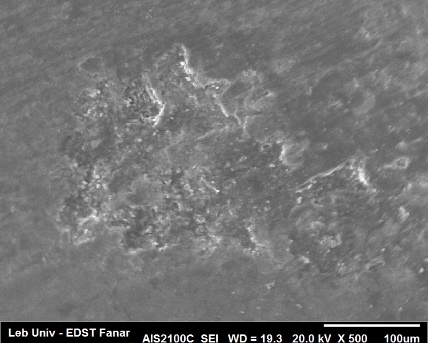 |
| 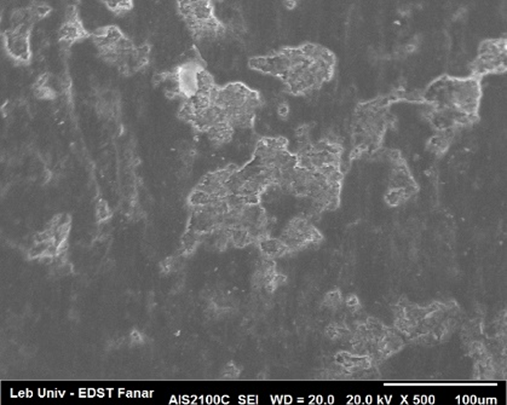 |  |  |
| Fig. 7D) Er:YAG 140mJ/20Hz | |  |
| 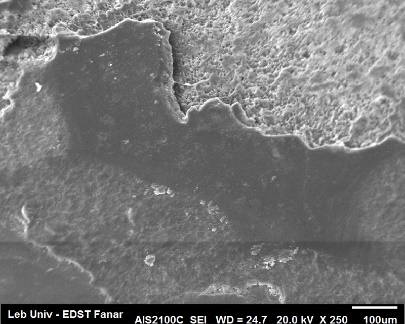 | 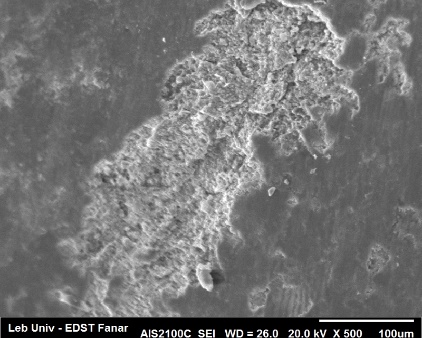 | 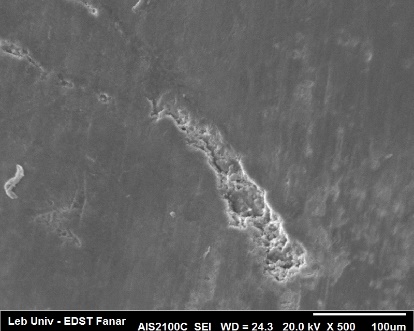 |
| Fig 7E) Er:YAG 80mJ/40Hz | |  |
| 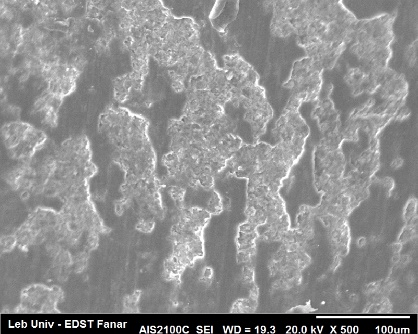 | 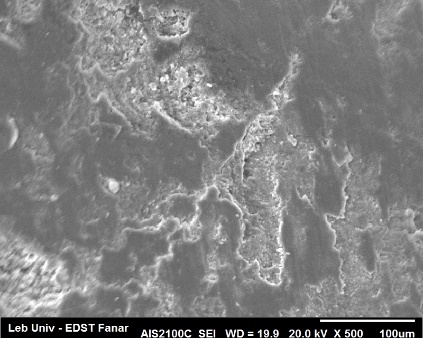 | 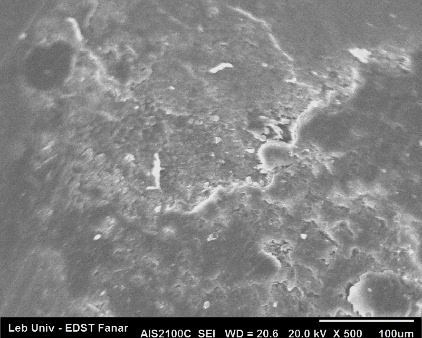 |
| Fig 7F) Er:YAG 100mJ/40Hz | |  |
| 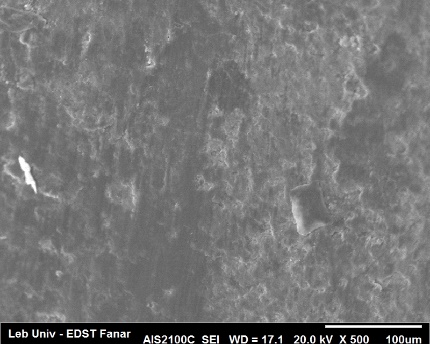 | 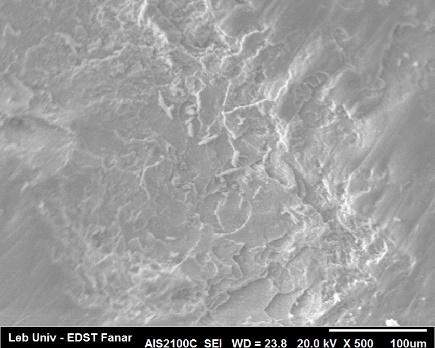 | 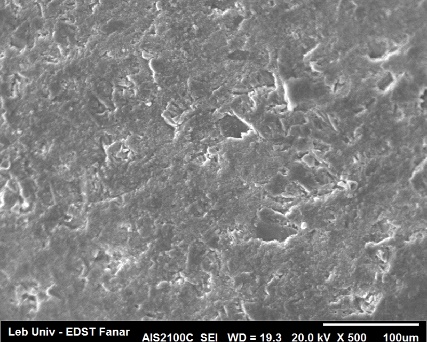 |
| Fig.7G) Er:YAG 120mJ/40Hz | |  |
| 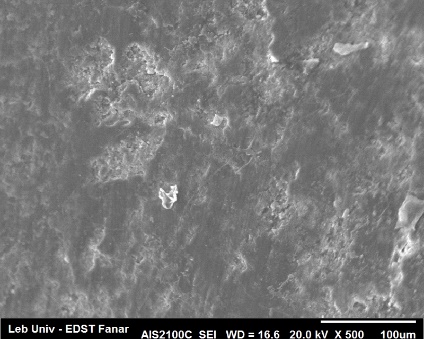 | 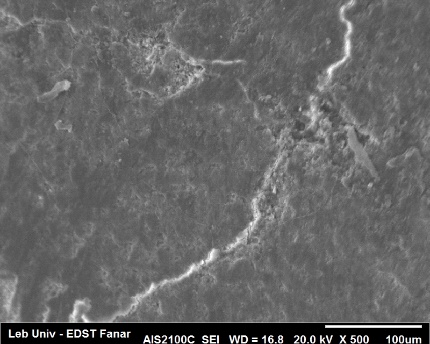 | 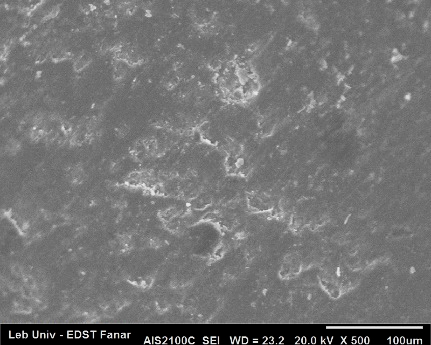 |
| Fig. 7H) Er:YAG 140mJ/40Hz | |  |
