# Supplementary figures and images for: Evaluation of Enamel Topography after Debonding Orthodontic Ceramic Brackets by Different Er,Cr:YSGG and Er:YAG Lasers Settings
Source: Dent J (Basel). 2020 Jan 9;8(1):6. doi: 10.3390/dj8010006 (PMC7175227; doi:10.3390/dj8010006)

Appendix of photos

| 3W/20Hz | 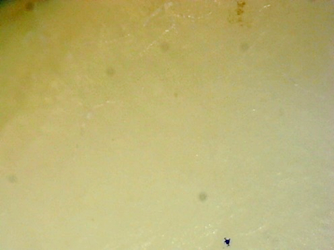 | 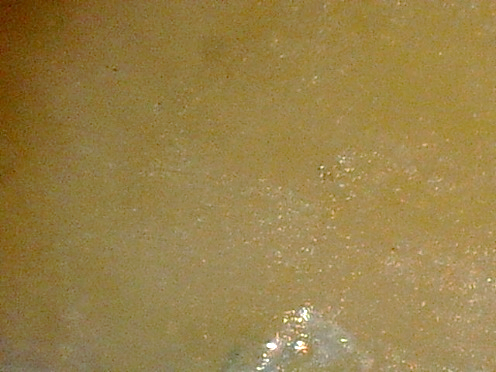 |
| --- | --- | --- |
| 3W/40Hz | 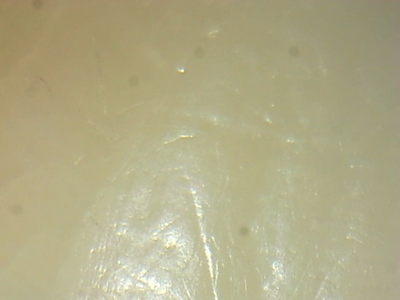 | 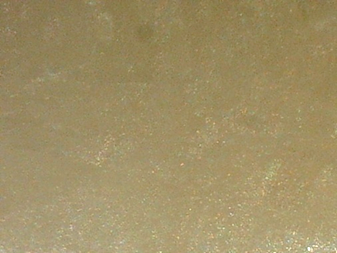 |
| 4W/20Hz | 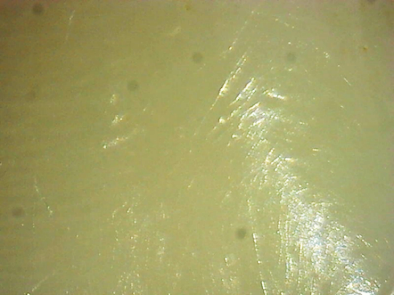 | 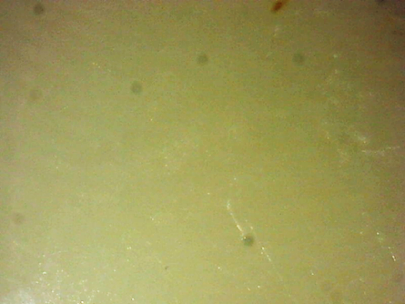 |
| 4W/40Hz | 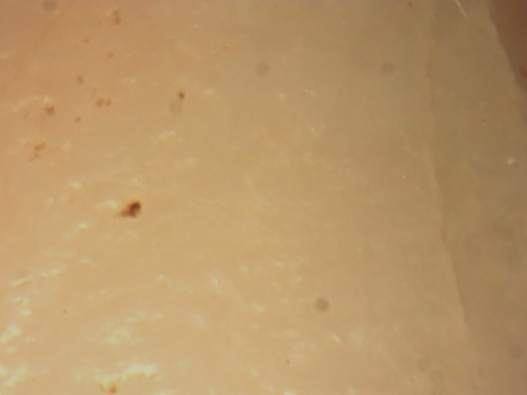 | 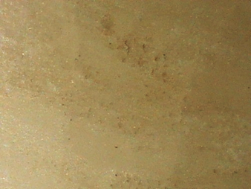 |
| 5W/20Hz | 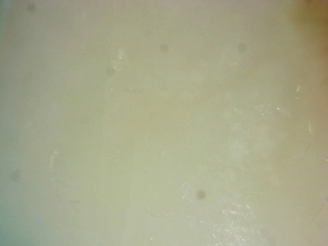 | 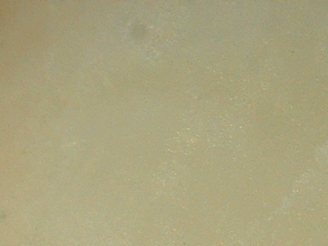 |
| 5W/40Hz | 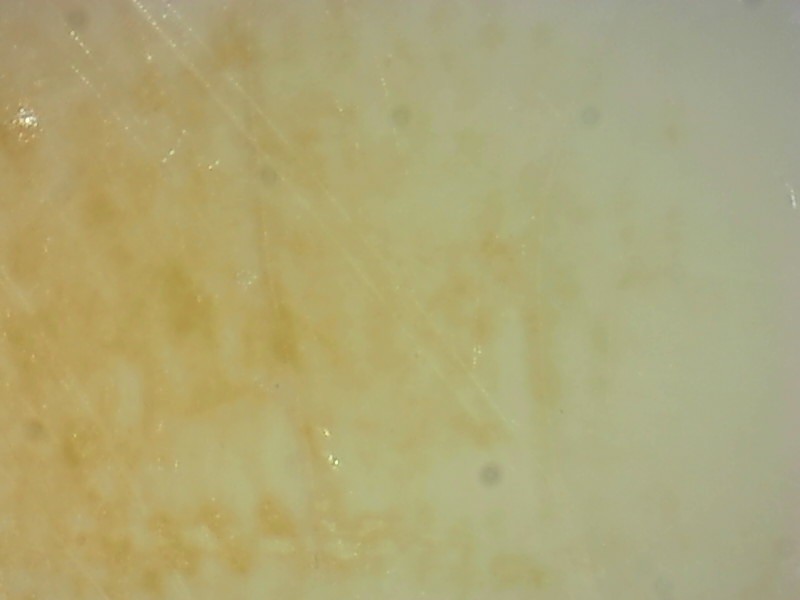 | 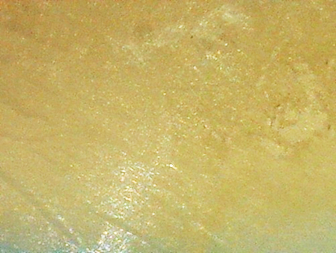 |

| 80mJ/20Hz | 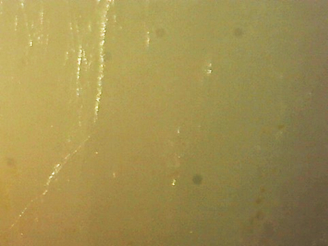 | 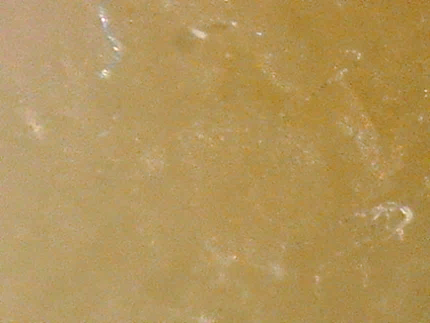 |
| --- | --- | --- |
| 80mJ/40Hz | 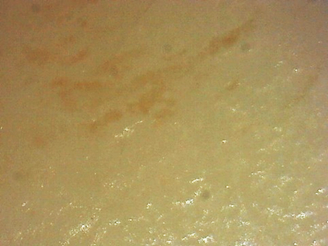 | 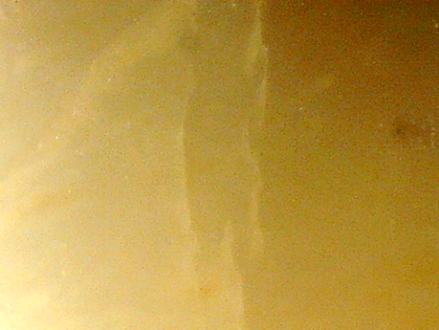 |
| 100mJ/20Hz | 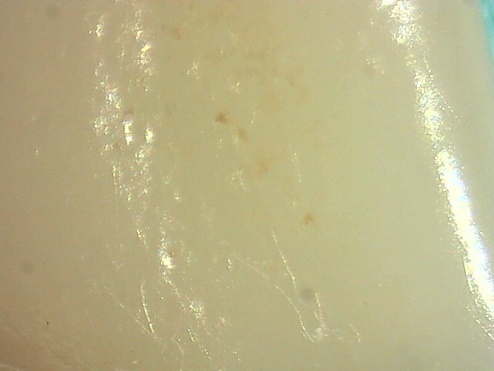 | 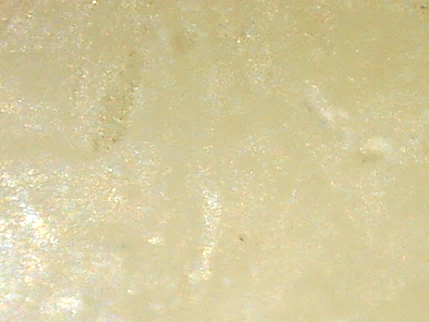 |
| 100mJ/40Hz | 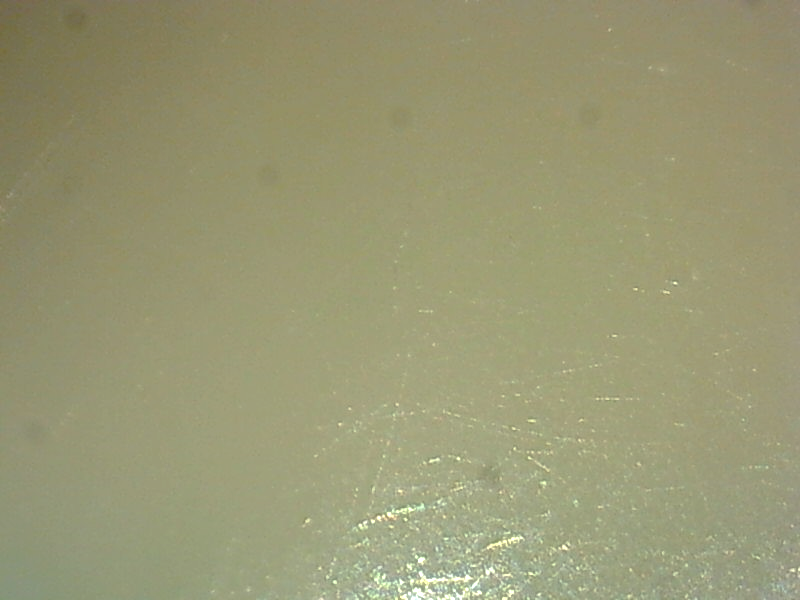 | 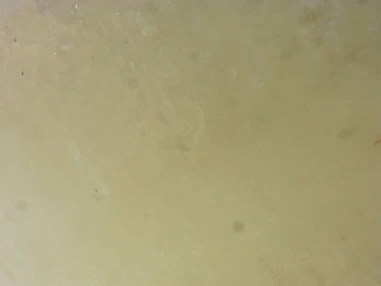 |
| 120mJ/20Hz | 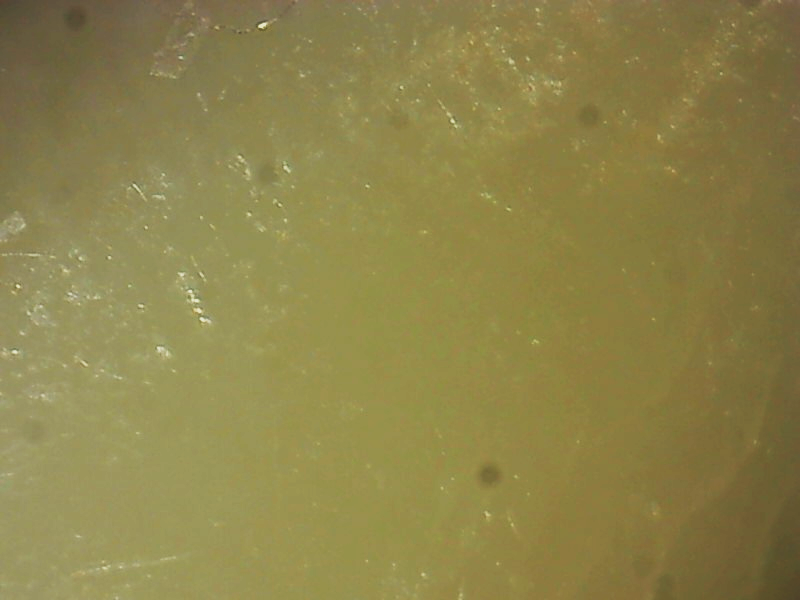 | 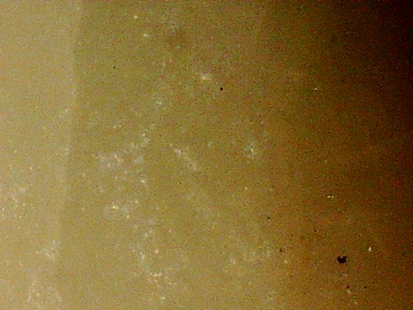 |
| 120mJ/40Hz | 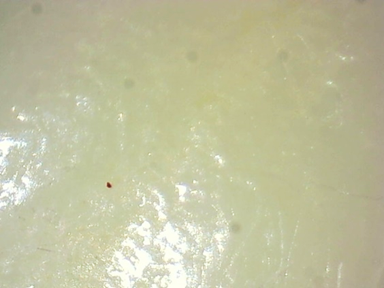 | 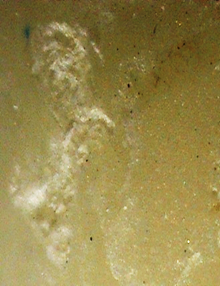 |
| 140mJ/20Hz | 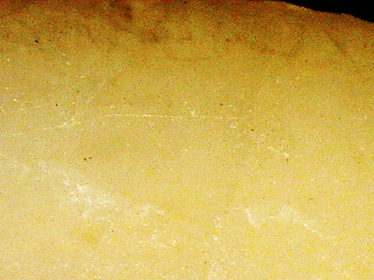 | 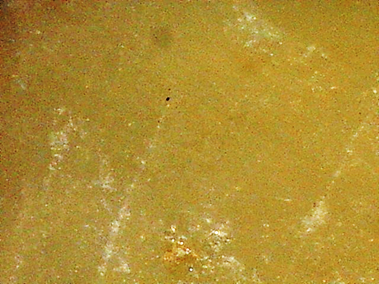 |
| 140mJ/40Hz | 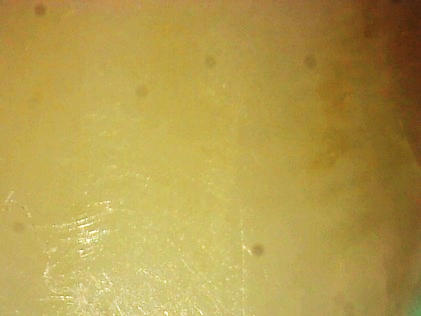 | 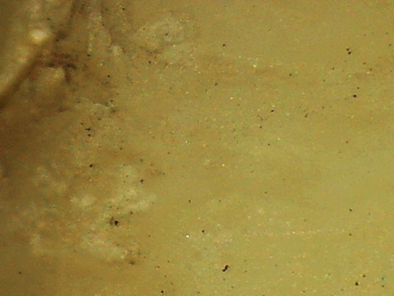 |

Supplement: Supplementary file 1 [file dentistry-08-00006-s001.zip › Supplementary file/S 1 (photos).docx]
